# Exploring perceived AI substitution in future accounting frameworks: the role of psychological trust, anxiety, and cognitive adaptability

**DOI:** 10.3389/fpsyg.2026.1875355

**Published:** 2026-07-20

**Authors:** Mingyu Yue

**Affiliations:** Faculty of Business, Hong Kong Polytechnic University, Kowloon, Hong Kong SAR, China

**Keywords:** accounting framework, AI anxiety, artificial intelligence, cognitive adaptability, psychological trust

## Abstract

Artificial intelligence (AI) is increasingly transforming accounting systems through automation, predictive analytics, intelligent auditing, and financial decision-support systems. While scholarly and professional debates frequently address whether AI may substitute human accountants, empirical evidence examining actual substitution remains limited. Existing studies have primarily focused on technological efficiency and adoption intentions, with insufficient attention given to the psychological factors shaping professionals' perceptions of AI's substitutive potential. This study therefore investigates how psychological trust, AI anxiety, and cognitive adaptability influence accounting professionals' perceptions regarding AI's potential to substitute selected accounting tasks within future accounting frameworks in China. A mixed-method research design integrating quantitative and qualitative approaches was employed. Primary data were collected from 512 accounting professionals across major Chinese cities using structured questionnaires. Partial Least Squares Structural Equation Modeling (PLS-SEM) was used to test the proposed relationships. Additionally, semi-structured interviews were conducted with 25 accounting professionals to contextualize and enrich the quantitative findings. The results indicate that psychological trust positively influences perceived AI task substitution, whereas AI anxiety negatively affects such perceptions. Cognitive adaptability emerged as a significant positive predictor and mediated the relationship between psychological trust and perceived AI substitution. Qualitative findings revealed that although participants perceived AI as having substantial potential to automate repetitive and analytical accounting activities, they consistently emphasized the continuing importance of human judgment, ethical reasoning, strategic interpretation, and interpersonal communication. The study contributes to AI-accounting and psychology literature by integrating psychological dimensions into understanding perceived AI substitution and suggests that future accounting frameworks are more likely to reflect human-AI complementarity rather than complete occupational replacement. These findings reflect accounting professionals' perceptions and expectations regarding AI substitution and can't be interpreted as evidence of AI's actual capability to perform accounting tasks or replace professional judgment.

## 1 Introduction

Artificial intelligence (AI) has emerged as one of the most disruptive technological innovations transforming the global business environment, particularly within accounting and financial management systems. The integration of AI technologies such as machine learning, robotic process automation (RPA), natural language processing, expert systems, and predictive analytics has fundamentally reshaped traditional accounting operations (Zhang et al., 2019a). Modern accounting systems increasingly rely on intelligent technologies for bookkeeping, auditing, fraud detection, financial forecasting, taxation, risk assessment, and regulatory compliance. These technologies have significantly enhanced operational efficiency, reduced human error, improved data processing capabilities, and accelerated financial decision-making processes (Davenport and Kirby, 2016).

The accounting profession has historically evolved alongside technological advancement. Earlier transitions from manual bookkeeping to computerized accounting systems revolutionized accounting practices by improving speed and accuracy. However, the current wave of AI-driven transformation differs substantially because AI technologies possess learning, predictive, and autonomous decision-making capabilities that imitate human cognitive functions. Consequently, the growing sophistication of AI systems has generated considerable debate regarding whether accounting professionals perceive AI as potentially serving as a substitute for selected accounting functions in the future (Sutton et al., 2016; Zhang et al., 2019b).

In recent years, China has emerged as a global leader in AI innovation and digital transformation. The Chinese government has strongly promoted AI development through national initiatives such as the “New Generation Artificial Intelligence Development Plan,” encouraging organizations to integrate intelligent systems across industries. Chinese enterprises increasingly adopt AI-driven accounting systems to improve financial efficiency, reduce labor costs, and strengthen competitive advantage. Major corporations in China are now utilizing intelligent financial platforms capable of automated invoice processing, smart auditing, predictive budgeting, and real-time financial analytics. The rapid expansion of digital accounting ecosystems in China makes it an ideal context for investigating perceptions regarding AI substitution in accounting practices (Li et al., 2023).

Despite the growing implementation of AI technologies, concerns regarding the complete replacement of human accountants remain highly controversial. Accounting is not solely a technical process involving numerical calculations and financial reporting. Rather, accounting also requires professional judgment, ethical reasoning, strategic thinking, emotional intelligence, contextual interpretation, communication skills, and stakeholder interaction (Tu et al., 2023). Human accountants frequently engage in complex decision-making processes that require moral evaluation, regulatory interpretation, and negotiation abilities that AI systems may struggle to replicate fully. Therefore, while AI can automate repetitive and rule-based accounting tasks, the extent to which it can substitute higher-order human cognitive functions remains uncertain (Moll and Yigitbasioglu, 2019).

Existing literature primarily focuses on the technological and organizational benefits of AI adoption in accounting. Previous studies have examined issues such as operational efficiency, cost reduction, technological readiness, auditing automation, and financial reporting accuracy (Kokina and Davenport, 2017; Wang et al., 2022). Several studies also investigate the role of AI in enhancing predictive financial analysis and fraud detection capabilities (Issa et al., 2016). However, the psychological dimensions influencing accountants' acceptance or resistance toward AI-enabled accounting systems remain significantly underexplored. This represents a critical research gap because technological adoption is not determined solely by system capability but also by users' psychological readiness, perceptions, emotions, and adaptability.

Recent psychological research suggests that responses to AI adoption extend beyond conventional technology acceptance considerations (Guo et al., 2024). Individuals evaluate AI through concerns related to algorithmic trust, explainability, professional identity, perceived job insecurity, and the anticipated redistribution of work between humans and intelligent systems (Glikson and Woolley, 2020; Logg et al., 2019; Brougham and Haar, 2018). In professional occupations such as accounting, these concerns are particularly salient because AI adoption challenges established expertise, occupational roles, and perceptions of professional value. Despite growing interest in the psychology of human–AI interaction, limited research has examined how these psychological processes shape accounting professionals' perceptions regarding AI's substitutive potential. Addressing this gap enables a shift from viewing AI acceptance solely as a technological issue toward understanding it as a process of occupational adaptation and identity negotiation within technologically transforming professions.

The rapid emergence of generative AI and large language models has substantially altered the nature of professional interactions with intelligent technologies. Unlike earlier forms of automation primarily designed to execute routine tasks, contemporary AI systems increasingly demonstrate capabilities related to content generation, analytical reasoning, decision support, and knowledge augmentation. Consequently, recent scholarship has shifted attention toward the psychological implications of AI integration, including algorithmic trust, explainability, AI anxiety, perceived job insecurity, professional identity, AI literacy, and human–AI collaboration (Choung et al., 2023; Kelly et al., 2023; Blease et al., 2023; Guo et al., 2024). Despite these developments, accounting research has largely remained focused on operational efficiency, technological readiness, and adoption intentions, with limited examination of how accounting professionals psychologically interpret AI-enabled occupational transformation. As a result, the literature provides insufficient understanding of how professionals perceive AI's substitutive potential in the context of rapidly evolving intelligent systems.

One of the most important psychological factors influencing AI adoption is psychological trust. Trust refers to individuals' willingness to rely on technology based on perceptions of competence, reliability, transparency, and predictability (Mayer et al., 1995). In accounting environments, professionals must trust AI-generated financial information before delegating critical accounting functions to intelligent systems (Zhang and Jiang, 2020). If accountants perceive AI systems as reliable and accurate, they are more likely to support the substitution of human tasks by AI technologies. Conversely, lack of trust may create resistance toward AI adoption despite technological superiority. Although trust has been widely studied in information systems research, limited empirical studies investigate how psychological trust shapes perceptions regarding AI substitution specifically within accounting frameworks. Another important yet underexplored factor is AI anxiety. AI anxiety refers to feelings of fear, uncertainty, discomfort, and insecurity associated with intelligent technologies (Wang and Wang, 2019). The increasing automation of accounting tasks has generated widespread concerns regarding job displacement, skill obsolescence, reduced professional autonomy, and technological dependency among accounting professionals. Employees may perceive AI as a threat to career stability and professional identity, resulting in psychological resistance toward AI-enabled systems. Previous research largely examines technological readiness and organizational implementation, while limited attention has been given to how AI anxiety influences perceptions regarding the future role of accountants in AI-driven financial environments (Jiang and Chen, 2018).

Furthermore, cognitive adaptability represents another significant psychological construct influencing technology acceptance. Cognitive adaptability refers to an individual's ability to adjust thinking patterns, learning strategies, and problem-solving approaches in response to changing technological environments (Haynie et al., 2010). In rapidly evolving digital accounting ecosystems, accounting professionals must continuously adapt to intelligent technologies, data analytics platforms, and automated financial systems. Individuals with high cognitive adaptability are more likely to embrace technological innovation and collaborate effectively with AI systems. However, existing accounting literature has rarely incorporated cognitive adaptability into AI adoption models, particularly within emerging economies such as China.

Another major gap in existing literature relates to methodological limitations. Most prior studies investigating AI adoption in accounting employ either conceptual discussions or quantitative-only approaches. Limited studies utilize mixed-method research designs combining quantitative modeling with qualitative insights. Quantitative approaches alone may fail to capture the deeper psychological experiences, fears, ethical concerns, and professional perceptions associated with AI-driven transformation in accounting professions. Therefore, integrating quantitative and qualitative methods can provide a more comprehensive understanding of how accounting professionals perceive AI substitution in practice. Additionally, prior studies often examine AI adoption using conventional technology acceptance theories such as the Technology Acceptance Model (TAM) and Unified Theory of Acceptance and Use of Technology (UTAUT), focusing primarily on perceived usefulness and ease of use (Venkatesh et al., 2003). Although prior studies debate whether AI may eventually substitute accountants, empirical evidence examining actual substitution remains scarce. Existing studies predominantly investigate technology adoption intentions, attitudes toward AI, or organizational readiness. Importantly, little is known about how accounting professionals perceive the substitutability of AI across accounting tasks and which psychological mechanisms shape these perceptions. Consequently, there remains a distinction between actual technological capability and professionals' beliefs regarding AI's potential role in future accounting work. The present study addresses this latter issue by investigating perceived rather than actual AI substitution.

The current study seeks to examine how accounting professionals perceive the potential of AI to substitute selected accounting tasks and to investigate how psychological trust, AI anxiety, and cognitive adaptability shape these perceptions in China. The study does not assess whether AI can objectively perform these tasks at a level equivalent to human accountants. The study employs a mixed-method research design integrating Partial Least Squares Structural Equation Modeling (PLS-SEM) with qualitative interviews to generate both statistical and contextual insights. By focusing on psychological dimensions, the study extends existing AI-accounting literature beyond technological efficiency perspectives and contributes toward understanding the human side of AI-enabled accounting transformation. However, the study does not evaluate actual accounting performance, decision quality, auditing accuracy, or the extent to which AI systems objectively replace accountants. Rather, it examines perceptions of AI substitution among accounting professionals exposed to increasingly digitalized accounting environments.

This study offers several important contributions. First, it contributes to the psychology and AI literature by advancing understanding of how accounting professionals interpret and respond to AI-enabled transformation through interconnected psychological processes involving algorithmic trust, automation-related anxiety, and occupational adaptation. By conceptualizing perceived AI substitution as a psychologically embedded response to changing professional boundaries, the study moves beyond conventional technology acceptance perspectives and highlights the roles of professional identity, perceived job insecurity, and human–AI collaboration in shaping professionals' perceptions of intelligent systems. Second, the study contributes methodologically by employing a mixed-method approach that combines PLS-SEM with qualitative inquiry, thereby providing both empirical rigor and deeper contextual insights into the psychological experiences underlying AI-enabled change. Third, it offers practical implications for policymakers, accounting firms, and AI developers by demonstrating that successful AI integration depends not only on technological capability but also on fostering trust, addressing automation-related concerns, enhancing AI literacy, and strengthening professionals' adaptive capacities to facilitate effective human–AI collaboration. Finally, the study contributes contextually by focusing on China, one of the world's fastest-growing AI-driven economies, where the rapid diffusion of intelligent accounting systems provides a valuable setting for understanding the psychological dimensions of technological transformation within professional occupations.

A central construct in the present study is Perceived AI Substitution (PAIS), which refers to accounting professionals' subjective beliefs regarding the extent to which artificial intelligence may potentially undertake selected accounting tasks traditionally performed by human accountants within future accounting environments. Importantly, PAIS does not represent actual technological substitution, objective AI capability, accounting performance, or occupational replacement. Rather, it captures professionals' perceptions, expectations, and interpretations concerning AI's future role in accounting work.

Although discussions concerning AI-enabled occupational substitution have become increasingly prominent in both academic and practitioner literature (Brynjolfsson and McAfee, 2014; Frey and Osborne, 2017), existing studies have primarily examined technology adoption, AI acceptance, automation readiness, and job insecurity rather than directly measuring perceived task substitution. Consequently, no widely accepted and psychometrically established scale currently exists that specifically measures perceived AI substitution within accounting contexts. To address this gap, the present study developed an exploratory measure designed to capture respondents' beliefs regarding AI's potential involvement in selected accounting activities. The scale could therefore be interpreted as an initial operationalization of an emerging construct rather than as a fully established measurement instrument. Consistent with recommendations for exploratory construct development (Churchill Jr, 1979; MacKenzie et al., 2011), the study undertook several procedures to establish conceptual clarity, content relevance, and preliminary psychometric adequacy.

## 2 Theoretical foundation and hypothesis development

### 2.1 Theoretical foundation

The present study is grounded in two major theoretical perspectives: the Technology Acceptance Model (TAM) and Trust Theory. While TAM explains how individuals evaluate and accept technological systems based on usefulness and usability perceptions, Trust Theory explains how confidence, reliability, and psychological assurance influence individuals' willingness to depend on AI systems. While TAM and Trust Theory provide important foundations for understanding technology acceptance, recent developments in AI psychology suggest that professionals' responses to intelligent systems are increasingly shaped by concerns extending beyond traditional perceptions of usefulness and reliability (Choung et al., 2024). The widespread deployment of generative AI has intensified debates surrounding transparency, algorithmic accountability, occupational disruption, and the evolving relationship between human expertise and machine intelligence (Blease et al., 2023). Accordingly, contemporary psychological perspectives are required to complement foundational theories and explain how professionals form perceptions regarding AI's potential role within knowledge-intensive occupations and interpret AI-enabled transformation.

#### 2.1.1 Technology Acceptance Model (TAM)

The Technology Acceptance Model (TAM), developed by (Davis 1989), is one of the most influential theories used to explain technology adoption behavior. TAM proposes that individuals' acceptance and usage of technological systems are primarily determined by two core constructs: perceived usefulness and perceived ease of use. Perceived usefulness refers to the degree to which individuals believe that a technology enhances their job performance, while perceived ease of use refers to the extent to which individuals believe that using the technology requires minimal effort (Davis, 1989). According to TAM, users are more likely to adopt technological systems when they perceive them as beneficial, efficient, and easy to operate. The theory further suggests that positive perceptions regarding technology influence behavioral intention, which subsequently determines actual usage behavior. Over the years, TAM has been extensively applied across multiple technological contexts, including information systems, e-commerce, healthcare technologies, educational technologies, and AI-enabled business systems (Venkatesh and Davis, 2000).

In the accounting profession, AI technologies such as robotic process automation, predictive analytics, intelligent auditing systems, and automated financial reporting platforms have significantly influenced accounting practices and workflows. Accounting professionals evaluate these systems based on their perceived ability to improve operational efficiency, reduce human error, increase analytical capability, and support decision-making processes. TAM provides a strong theoretical explanation for understanding why accounting professionals may accept or resist AI-enabled accounting systems. Within the context of the present study, psychological trust and cognitive adaptability can be interpreted through TAM principles. When accounting professionals perceive AI systems as reliable and beneficial, they are more likely to recognize the usefulness of AI technologies in performing accounting tasks. Trust in AI systems increases perceptions regarding system efficiency, transparency, and accuracy, thereby strengthening acceptance toward perceived AI substitution in accounting functions. Similarly, cognitive adaptability enhances professionals' ability to learn and utilize AI technologies effectively, thereby reducing complexity perceptions and increasing perceived ease of use.

Conversely, AI anxiety negatively influences technology acceptance by generating fear, uncertainty, and resistance toward AI systems. Individuals experiencing high levels of anxiety may perceive AI technologies as threatening, difficult to understand, or disruptive to professional stability. Such perceptions reduce willingness to adopt AI-enabled systems, even when technological benefits are evident. Therefore, TAM provides an important framework for explaining how psychological factors shape AI adoption behavior within accounting environments. The relevance of TAM in the current study is particularly important because accounting professionals in China are increasingly exposed to intelligent financial systems and digital accounting infrastructures. The rapid implementation of AI-driven accounting technologies requires professionals to evaluate whether they perceive these systems as having the potential to undertake tasks traditionally performed by humans. TAM explains how accountants' perceptions regarding usefulness, efficiency, and adaptability influence their acceptance of perceived AI substitution within future accounting frameworks. Furthermore, previous studies have successfully applied TAM to examine AI adoption and intelligent technologies in professional environments. (Venkatesh and Davis 2000) extended TAM by emphasizing the role of social influence and cognitive processes in technology adoption behavior. Similarly, accounting technology studies demonstrate that professionals are more likely to adopt intelligent systems when they perceive them as beneficial for improving work performance and reducing workload (Sutton et al., 2016).

The present study extends TAM by incorporating psychological dimensions such as trust, anxiety, and cognitive adaptability into the AI-accounting adoption framework. While traditional TAM studies primarily focus on technological perceptions, this study recognizes that emotional and cognitive responses toward AI systems also significantly influence acceptance behavior. Therefore, TAM serves as a foundational theory explaining how accounting professionals evaluate AI systems and form perceptions regarding AI's potential role within technologically evolving accounting environments.

Beyond traditional technology acceptance perspectives, recent accounting research increasingly conceptualizes AI as a governance and intelligent control mechanism operating within organizational accounting systems. (Bonelli 2026) argues that AI-enabled accounting transformation involves a shift from manual accounting practices toward intelligent accounting architectures characterized by automated controls, predictive analytics, continuous monitoring, and enhanced risk management capabilities. This perspective is particularly relevant to the present study because accounting professionals' perceptions regarding AI substitution are shaped not only by technological functionality but also by beliefs regarding governance reliability, accountability, risk management effectiveness, and the legitimacy of algorithmic decision-making. Consequently, professionals' trust in AI systems, anxiety regarding technological transformation, and capacity for cognitive adaptation may substantially influence perceptions concerning the appropriate role of intelligent systems within future accounting frameworks.

#### 2.1.2 Trust Theory

Trust Theory provides the second major theoretical foundation supporting the present study. Trust is a fundamental psychological mechanism influencing individuals' willingness to depend on technologies, organizations, and systems under conditions of uncertainty and risk. (Mayer et al. 1995) define trust as the willingness of an individual to become vulnerable to the actions of another party based on expectations regarding competence, reliability, integrity, and benevolence. In technology-related contexts, trust plays a critical role in shaping users' acceptance of intelligent systems, especially when technologies perform complex or autonomous tasks. AI systems operate through algorithmic decision-making, machine learning, and predictive analytics, often producing outputs beyond users' direct understanding. Consequently, individuals must psychologically trust AI systems before relying on them in important professional activities.

Within accounting environments, trust becomes particularly significant because accounting tasks involve financial accuracy, ethical responsibility, legal compliance, and strategic decision-making. Accounting professionals must develop confidence that AI systems can appropriately support activities such as financial reporting, fraud detection, transaction processing, and data management. If professionals perceive AI systems as transparent, competent, and dependable, they are more likely to support AI integration and perceive greater potential for AI substitution in accounting operations. The current study specifically examines psychological trust as a central determinant of perceptions regarding AI substitution in accounting tasks. Trust Theory suggests that when individuals perceive technologies as reliable and beneficial, they develop confidence in using and depending on those systems. In contrast, lack of trust generates skepticism, resistance, and fear toward technological systems.

Trust Theory is highly relevant in the context of AI-enabled accounting because AI systems often operate autonomously, reducing direct human intervention in financial processes. Professionals may become concerned about algorithmic bias, ethical accountability, data manipulation, and system errors. Therefore, trust becomes essential for facilitating AI acceptance in accounting frameworks. The relationship between trust and cognitive adaptability is also theoretically supported by Trust Theory. Individuals who trust AI systems are more likely to engage positively with technological learning processes and adapt their cognitive strategies to technological environments. Trust reduces uncertainty and enhances confidence in experimenting with new technologies, thereby improving cognitive adaptability. Conversely, low trust may increase resistance toward technological learning and reduce adaptability. Moreover, Trust Theory explains the negative influence of AI anxiety on AI adoption. Anxiety emerges when individuals perceive technologies as unpredictable, threatening, or uncontrollable. Professionals experiencing AI anxiety may fear job displacement, reduced professional relevance, and technological dependency. These fears weaken trust in AI systems and reduce willingness to accept perceived AI substitution within accounting professions.

Previous studies demonstrate that trust significantly influences AI adoption behavior across various organizational settings. Research shows that trust enhances acceptance of intelligent decision-support systems, automated technologies, and machine-learning applications (Glikson and Woolley, 2020). Similarly, studies in accounting and auditing contexts indicate that professionals' trust in intelligent systems determines their willingness to rely on AI-generated outputs (Issa et al., 2016). The integration of Trust Theory into the present study is particularly important because AI adoption within accounting is not merely a technological issue but also a psychological and ethical issue. Accounting professionals must psychologically trust intelligent systems before accepting perceptions that AI may undertake selected accounting activities. Therefore, Trust Theory provides a robust explanation for understanding how psychological confidence, reliability perceptions, and emotional responses influence perceptions regarding AI-enabled accounting transformation.

The integration of TAM and Trust Theory provides a comprehensive framework for understanding perceptions of AI substitution in accounting. TAM explains how accounting professionals evaluate AI systems based on usefulness and adaptability, whereas Trust Theory explains how psychological confidence and emotional assurance influence willingness to depend on AI systems.

#### 2.1.3 Psychological perspective on human–AI collaboration and occupational adaptation

Recent advances in the psychology of artificial intelligence suggest that professionals' responses to AI extend beyond traditional technology acceptance considerations such as usefulness and ease of use. Rather than evaluating AI solely as a technological artifact, individuals interpret intelligent systems through psychological processes involving trust in algorithms, explainability, professional identity, perceived job insecurity, AI literacy, and expectations regarding future collaboration between humans and machines. These factors become particularly salient in professional occupations such as accounting, where expertise, ethical judgment, autonomy, and occupational identity constitute central elements of professional practice. Consequently, understanding perceptions regarding AI substitution requires moving beyond conventional adoption frameworks to consider how individuals psychologically negotiate technological disruption within their professional environments.

One of the most influential developments in recent psychology literature concerns the concepts of algorithm aversion and algorithm appreciation (Chu et al., 2021). (Dietvorst et al. 2015) demonstrated that individuals frequently become reluctant to rely on algorithmic systems after observing even minor errors, despite evidence that algorithms often outperform human decision-makers. This tendency, referred to as algorithm aversion, reflects concerns regarding the perceived inflexibility, opacity, and contextual limitations of algorithmic systems. Conversely, subsequent research suggests that individuals may display algorithm appreciation when they perceive AI systems as highly competent, transparent, and capable of enhancing decision quality (Logg et al., 2019; Jiang et al., 2022). These contrasting psychological responses indicate that acceptance of AI is not merely a rational evaluation of performance but also depends on subjective interpretations of algorithmic reliability and legitimacy. Within accounting contexts, professionals may simultaneously acknowledge AI's perceived efficiency in processing financial information while remaining hesitant to fully rely on its outputs for tasks involving ambiguity, professional skepticism, or contextual judgment.

Closely related to these perspectives is the growing emphasis on explainability and trust in AI. Explainability refers to the extent to which users understand how AI systems generate recommendations and arrive at decisions. Research has consistently shown that opaque or “black-box” algorithms tend to undermine trust because users struggle to comprehend the rationale underlying automated outputs (Glikson and Woolley, 2020). Explainable AI, in contrast, enhances users' perceptions of competence, predictability, and accountability, thereby increasing their willingness to collaborate with intelligent systems (Jöhnk et al., 2021). In accounting environments, explainability assumes particular importance because professionals operate under strict regulatory requirements and are expected to justify financial decisions to clients, auditors, regulators, and other stakeholders. Accounting professionals may therefore be reluctant to support perceived AI-enabled task substitution unless intelligent systems produce outputs that are transparent, interpretable, and defensible. Psychological trust in AI thus reflects not only beliefs regarding technical competence but also confidence in the explainability and accountability of algorithmic recommendations.

Another emerging psychological concern relates to professional identity and perceived job insecurity. Professional identity refers to individuals' understanding of who they are within their occupational roles, encompassing their values, expertise, and perceptions of professional worth (Song et al., 2026). The introduction of AI technologies may challenge these identities by altering traditional role boundaries and redistributing tasks previously associated with professional expertise. (Brougham and Haar 2018) argued that intelligent technologies often generate concerns regarding future employability, diminished occupational relevance, and uncertainty about career trajectories. Such concerns may be especially pronounced in accounting because routine accounting functions, including bookkeeping, reconciliation, and transactional processing, have become increasingly automated. Professionals may therefore interpret AI not only as a productivity-enhancing technology but also as a symbolic threat to the uniqueness and value of their expertise. These perceptions can contribute to AI anxiety and resistance toward perceived AI substitution, even when individuals recognize the perceived operational benefits of intelligent systems.

The psychology of human–AI collaboration offers an alternative perspective to narratives emphasizing technological replacement. Rather than conceptualizing humans and AI as competitors, recent scholarship increasingly views AI as a collaborative partner capable of augmenting human capabilities (Dellermann et al., 2019; Chung et al., 2015). The emergence of generative AI has further strengthened arguments supporting augmentation rather than complete replacement (Yang et al., 2020). Recent evidence suggests that professionals increasingly view AI as a collaborative resource capable of enhancing productivity and analytical capability while preserving uniquely human competencies involving ethical reasoning, contextual interpretation, and interpersonal engagement (Dwivedi et al., 2023). In professional contexts, intelligent systems are often perceived as being particularly useful for processing large volumes of structured information, identifying patterns, and generating predictive insights, whereas humans are generally viewed as retaining advantages in ethical reasoning, contextual interpretation, creativity, empathy, and stakeholder communication. From this perspective, technological transformation involves perceived redistribution of responsibilities between humans and intelligent systems rather than the complete elimination of professional roles. Within accounting, professionals may perceive AI as having the potential to undertake selected routine and analytical tasks while simultaneously increasing the importance of accountants' strategic, advisory, and judgment-oriented functions. Consequently, perceptions regarding AI substitution may coexist with expectations of increased human–AI interdependence.

A further psychological construct receiving growing attention is AI literacy and occupational adaptation. AI literacy refers to individuals' understanding of AI capabilities, limitations, and appropriate applications, enabling them to critically evaluate and effectively engage with intelligent systems (Ng et al., 2021). Employees possessing higher levels of AI literacy are generally more capable of distinguishing between tasks suitable for automation and those requiring human expertise (Zhang et al., 2021). Such understanding facilitates realistic expectations regarding AI implementation and reduces unnecessary fears associated with technological uncertainty. Similarly, occupational adaptation emphasizes individuals' capacity to redefine their professional roles, acquire new competencies, and adjust to evolving work environments. Rather than merely learning to use new technologies, occupational adaptation involves identity reconstruction, continuous learning, and the development of resilience in response to technological disruption (Savickas, 2013). In accounting professions undergoing rapid digital transformation, adaptive individuals are more likely to perceive AI as an opportunity for professional evolution rather than as an existential threat.

Drawing on these emerging psychological perspectives, the present study argues that accounting professionals' perceptions of AI substitution are shaped by broader processes of occupational interpretation and adaptation rather than by evaluations of technological capability alone. Psychological trust reflects confidence in the competence, transparency, and explainability of AI systems, thereby fostering greater willingness to engage with intelligent technologies. In contrast, AI anxiety encompasses concerns related to professional identity threats, job insecurity, and uncertainty about future occupational relevance. Cognitive adaptability represents the capacity through which professionals respond to technological disruption by redefining their roles, acquiring new competencies, and developing collaborative approaches to working alongside intelligent systems. By integrating these psychological dimensions, the present study extends traditional technology acceptance frameworks and offers a more nuanced understanding of how accounting professionals interpret and respond to AI-driven transformation. Accordingly, perceived AI substitution is conceptualized not as a judgment regarding AI's actual capability to replace accounting professionals or perform accounting tasks objectively, but as a psychologically embedded perception concerning AI's potential role within evolving occupational boundaries in increasingly AI-enabled accounting environments.

#### 2.1.4 AI-enabled accounting transformation and intelligent accounting systems

Recent developments in artificial intelligence have accelerated the transformation of accounting from a predominantly manual and transaction-oriented profession toward intelligent, data-driven, and algorithmically supported accounting ecosystems. Contemporary accounting systems increasingly integrate machine learning, robotic process automation (RPA), natural language processing, predictive analytics, intelligent auditing tools, and continuous monitoring technologies that automate routine accounting processes while enhancing analytical and decision-support capabilities (Moll and Yigitbasioglu, 2019; Kokina and Davenport, 2017). Consequently, accounting research has shifted from examining technology adoption alone toward understanding how intelligent systems reshape professional roles, governance structures, organizational controls, and the nature of accounting work itself.

The concept of intelligent accounting systems has emerged as a central theme within recent accounting literature. Unlike traditional computerized accounting systems that primarily support data recording and reporting, intelligent accounting systems possess the capacity to learn from historical data, identify anomalies, generate predictive insights, support compliance monitoring, and assist in strategic decision-making (Sutton et al., 2016; Issa et al., 2016). These developments have expanded the role of AI from a transactional automation tool to an integral component of organizational governance, risk management, and internal control systems.

Recent evidence suggests that AI increasingly functions as an intelligent control mechanism within organizations. (Bonelli 2026) demonstrated that AI-enabled accounting systems contribute significantly to governance effectiveness, risk monitoring, compliance management, and organizational control processes. The study highlighted how organizations are transitioning from manual accounting practices toward intelligent accounting architectures characterized by automated controls, predictive monitoring, and real-time risk assessment. Importantly, this transformation extends beyond operational efficiency and involves fundamental changes in accountability structures, professional responsibilities, and trust relationships between human professionals and algorithmic systems. The findings indicate that successful implementation of intelligent accounting systems depends not only on technological capabilities but also on professionals' confidence in the transparency, reliability, and governance legitimacy of AI-enabled controls.

The growing integration of AI into accounting has also intensified scholarly interest in algorithmic trust and explainability. Accounting professionals frequently operate in environments characterized by regulatory scrutiny, fiduciary responsibility, and high accountability requirements. Under such conditions, reliance on AI-generated outputs depends heavily on trust in algorithmic recommendations and confidence in the explainability of intelligent systems (Glikson and Woolley, 2020; Jöhnk et al., 2021). Recent studies suggest that professionals are more willing to accept AI-supported decisions when intelligent systems provide transparent, interpretable, and auditable explanations for their outputs (Choung et al., 2023; Blease et al., 2023). In accounting contexts, explainability becomes particularly important because professionals remain accountable for financial reporting outcomes even when AI contributes substantially to data analysis and decision support.

Another important stream of recent literature focuses on governance and risk implications arising from AI-enabled accounting transformation. AI systems increasingly support fraud detection, anomaly identification, compliance monitoring, predictive risk assessment, and continuous auditing functions (Appelbaum et al., 2017; Kokina and Davenport, 2017). While these capabilities improve organizational oversight and operational efficiency, they also introduce concerns regarding algorithmic bias, data quality, cybersecurity risks, accountability gaps, and excessive dependence on automated decision-making. Consequently, researchers have emphasized the need to balance technological efficiency with governance safeguards, human oversight, and professional accountability mechanisms (Bonelli, 2026; Dwivedi et al., 2023).

Recent scholarship further argues that AI-enabled accounting transformation should be understood as a process of professional and occupational change rather than simple technological substitution. As intelligent systems increasingly automate routine and rule-based accounting activities, the professional role of accountants is evolving toward higher-order activities involving strategic analysis, judgment, stakeholder communication, ethical evaluation, and advisory functions (Moll and Yigitbasioglu, 2019; Tu et al., 2023). Rather than eliminating accounting professions entirely, AI appears to be reshaping the boundaries between machine-performed tasks and human expertise. This transition has generated growing interest in how accounting professionals psychologically interpret AI-enabled transformation and how perceptions of trust, anxiety, professional identity, and adaptability influence acceptance of intelligent accounting systems.

Although recent studies have significantly advanced understanding of intelligent accounting systems, governance mechanisms, and AI-enabled organizational transformation, important gaps remain. Existing accounting research has predominantly focused on technological capabilities, operational benefits, continuous auditing, fraud detection, and organizational implementation outcomes (Issa et al., 2016; Appelbaum et al., 2017; Bonelli, 2026). Comparatively less attention has been devoted to the psychological processes through which accounting professionals interpret AI-enabled transformation and form perceptions regarding AI's potential to substitute selected accounting tasks. In particular, limited empirical evidence exists regarding how psychological trust, AI anxiety, and cognitive adaptability jointly influence professionals' perceptions concerning the evolving boundaries between human expertise and intelligent accounting systems. Addressing this gap is increasingly important because the success of intelligent accounting systems ultimately depends not only on technological sophistication but also on professionals' willingness to trust, adapt to, and collaborate with AI-enabled accounting technologies.

**Figure 1:**
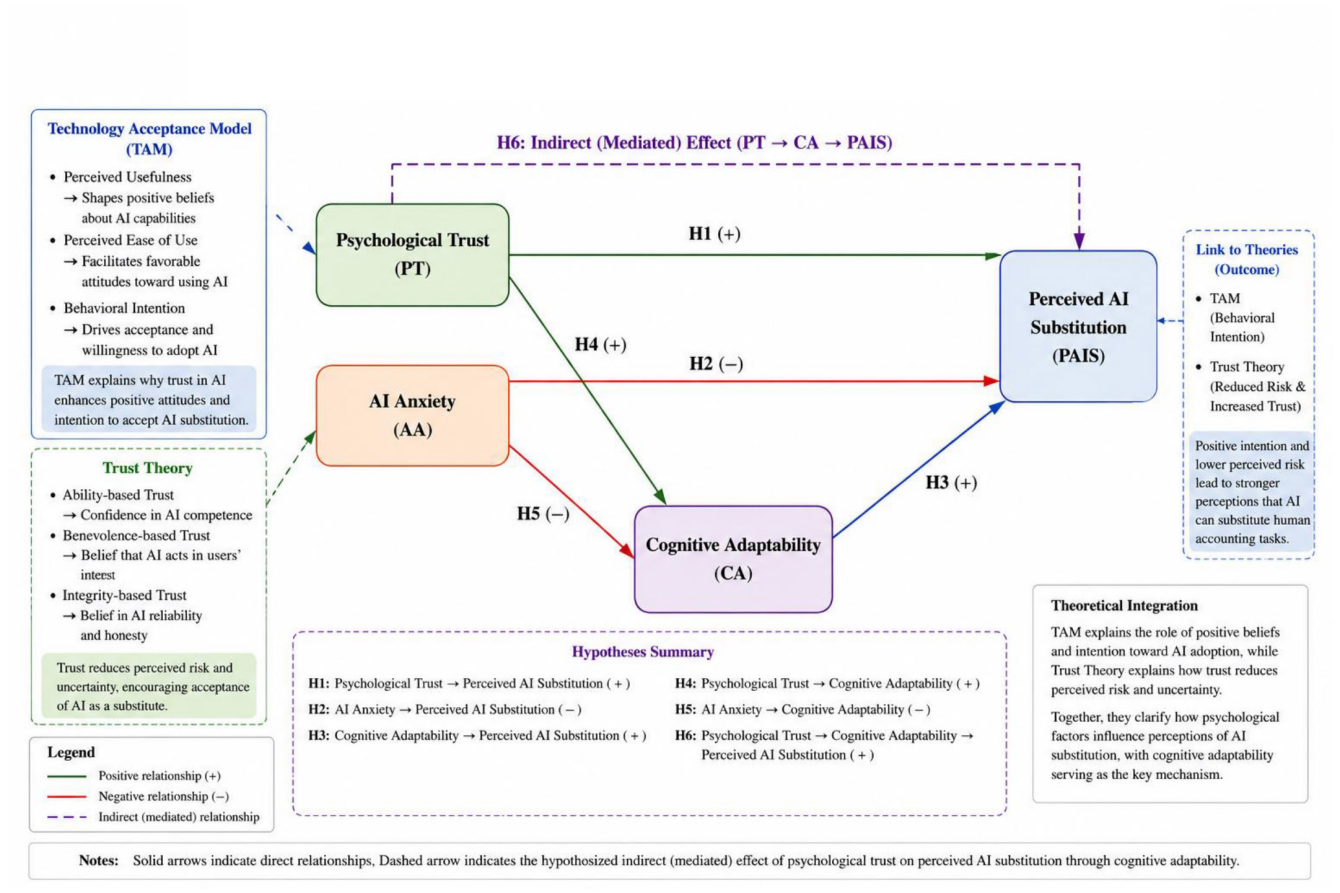
Proposed conceptual framework. Grounded in the Technology Acceptance Model (TAM) and Trust Theory, the framework explains how psychological trust and AI anxiety shape accounting professionals' perceptions of AI substitution, with cognitive adaptability acting as a key mechanism and mediator.

Figure 1 presents the proposed conceptual framework of the study, illustrating the hypothesized relationships among psychological trust, AI anxiety, cognitive adaptability, and perceived AI substitution within future accounting frameworks. Grounded in the Technology Acceptance Model (TAM) and Trust Theory, the framework posits that psychological trust positively influences accounting professionals' perceptions regarding AI's potential to substitute selected accounting tasks (H1), whereas AI anxiety exerts a negative influence on such perceptions (H2). The model further proposes that cognitive adaptability positively affects perceived AI substitution (H3) and serves as a key psychological mechanism shaped by both psychological trust and AI anxiety. Specifically, psychological trust is expected to enhance cognitive adaptability (H4), while AI anxiety is anticipated to weaken individuals' adaptive capacity toward AI-enabled environments (H5). In addition, the framework advances the proposition that cognitive adaptability mediates the relationship between psychological trust and perceived AI substitution (H6), suggesting that trusted AI systems promote acceptance partly by strengthening professionals' ability to adjust cognitively to technological change. Collectively, the framework conceptualizes perceived AI substitution not as a purely technological judgment but as a psychologically embedded response to the evolving boundaries between human expertise and intelligent systems in contemporary accounting practice.

### 2.2 Hypothesis development

#### 2.2.1 Psychological trust and AI substitution perception

Psychological trust is regarded as one of the most critical determinants influencing the acceptance and integration of artificial intelligence (AI) systems within professional environments. Trust generally refers to an individual's willingness to rely on another entity based on positive expectations regarding reliability, competence, integrity, and predictability (Mayer et al., 1995). In technology-oriented contexts, psychological trust reflects users' confidence that technological systems will perform accurately, consistently, securely, and ethically. As AI technologies become increasingly integrated into accounting operations, trust becomes essential in determining whether accounting professionals perceive AI as having the potential to substitute selected human accounting tasks. The accounting profession involves highly sensitive financial activities such as auditing, taxation, budgeting, financial reporting, compliance monitoring, and fraud detection. These activities require accuracy, confidentiality, transparency, and regulatory compliance. AI-enabled accounting systems are increasingly used in activities such as data processing, transaction matching, anomaly detection, and analytical support through machine learning algorithms, robotic process automation, and predictive analytics. However, professionals' perceptions regarding AI's potential to substitute selected accounting tasks depend significantly on whether they psychologically trust these intelligent systems.

Trust becomes particularly important in AI-enabled environments because AI technologies often function autonomously with limited human intervention. Unlike traditional computerized accounting software, AI systems continuously learn from data, make predictions, identify patterns, and support decision-making processes independently. This autonomous nature creates uncertainty among users regarding system transparency, accountability, ethical reliability, and algorithmic accuracy. Consequently, accounting professionals must develop psychological confidence in AI systems before delegating critical accounting responsibilities to them (Glikson and Woolley, 2020; Luo et al., 2018; Pallant, 2020). According to Trust Theory, individuals are more likely to depend on systems they perceive as competent, predictable, and reliable (Mayer et al., 1995). In accounting contexts, trust in AI systems develops when professionals believe that intelligent technologies can reliably support financial data processing, anomaly identification, and decision-support activities. AI systems capable of generating reliable accounting outputs are more likely to gain acceptance among accounting professionals because trust reduces perceptions of technological risk and uncertainty. The Technology Acceptance Model (TAM) further explains the importance of trust in technological adoption behavior. TAM proposes that individuals adopt technologies when they perceive them as useful and easy to use (Davis, 1989). Psychological trust strengthens perceptions of usefulness because professionals who trust AI systems are more likely to believe that these technologies can support accounting activities and reduce routine work demands. When accountants trust AI systems, they are more likely to perceive AI as having the potential to perform certain accounting tasks traditionally completed by humans.

In modern accounting environments, trust is especially relevant because accounting professionals often rely on AI-generated recommendations and automated financial analyses for strategic decision-making. AI-driven systems are often perceived as useful for processing large financial datasets and supporting activities such as real-time reporting, predictive forecasting, and intelligent auditing. When professionals trust these capabilities, they become more open to accepting perceived AI substitution in repetitive and analytical accounting functions.

Previous empirical studies support the positive relationship between trust and AI adoption. (Glikson and Woolley 2020) found that trust significantly influences individuals' willingness to rely on AI technologies across organizational settings. The relationship is also consistent with explainability research suggesting that professionals are more likely to rely on algorithmic systems when they perceive them as transparent and understandable. Similarly, (Choung et al. 2023) reported that users who perceive AI systems as reliable and transparent demonstrate higher acceptance of AI-enabled decision-making systems. In financial and accounting contexts, trust in intelligent technologies positively affects reliance on automated auditing systems and predictive financial tools (Issa et al., 2016). Moreover, trust reduces psychological resistance toward technological transformation. Accounting professionals often experience uncertainty regarding AI implementation because intelligent technologies may alter traditional work structures and professional responsibilities. Trust mitigates such concerns by creating confidence that AI systems are designed to support rather than undermine professional accounting functions. Professionals who trust AI technologies are more likely to view AI as a collaborative and beneficial tool rather than a threat to professional identity.

The role of trust becomes even more significant in China due to the rapid digital transformation of Chinese enterprises and governmental support for AI development. China has emerged as one of the world's leading AI-driven economies, with organizations increasingly implementing intelligent financial systems and automated accounting platforms. As accounting firms and corporations integrate AI into financial operations, professionals' trust in intelligent systems becomes essential for successful technological transition. Chinese accounting professionals who trust AI systems are more likely to perceive intelligent technologies as having the potential to handle routine accounting functions such as bookkeeping, invoice processing, transaction verification, and financial reconciliation (Chung et al., 2020). Trust may encourage professionals to place greater confidence in AI-generated insights for financial forecasting, fraud detection, and strategic planning. Consequently, trust facilitates positive attitudes toward perceived AI substitution within accounting frameworks. In addition, trust may influence professionals' perceptions regarding ethical reliability and data security in AI systems. Accounting information is highly confidential and sensitive, making data integrity and security fundamental concerns within AI-enabled accounting environments. Professionals who trust AI technologies are more likely to believe that intelligent systems can securely manage financial information and comply with ethical and regulatory standards. This perception strengthens professionals' willingness to consider AI involvement in selected accounting tasks.

The relationship between psychological trust and AI substitution perception is also supported by social exchange perspectives, which suggest that individuals engage positively with systems when expected benefits outweigh perceived risks. Trusted AI systems reduce uncertainty and increase confidence regarding technological outcomes. Consequently, professionals are more likely to support perceived AI substitution when they perceive intelligent technologies as accurate, beneficial, and dependable. Furthermore, trust enhances professionals' openness toward technological experimentation and learning. Individuals who trust AI systems are generally more willing to explore intelligent technologies, participate in AI-related training programs, and integrate AI tools into daily accounting activities. This positive orientation further strengthens perceptions that AI has the potential to substitute certain repetitive accounting tasks. Recent studies indicate that trust in AI increasingly depends on perceptions of explainability, accountability, and the ability of users to understand how intelligent systems generate recommendations (Choung et al., 2023; Blease et al., 2023; Wang et al., 2024). Particularly in high-stakes professions such as accounting, professionals are more likely to rely on AI when they perceive algorithmic outputs as transparent, defensible, and aligned with professional standards.

Recent evidence from intelligent accounting systems further reinforces the importance of trust in AI-enabled accounting environments. (Bonelli 2026) found that governance effectiveness within intelligent accounting systems depends heavily on users' confidence in algorithmic controls, predictive analytics, and automated decision-support mechanisms. Professionals are more willing to accept AI involvement in accounting activities when intelligent systems are perceived as transparent, accountable, and aligned with organizational governance requirements. Accordingly, trust not only supports technological acceptance but also facilitates confidence in AI's ability to participate in accounting control and decision-support functions.

However, despite the importance of trust, prior accounting literature has largely focused on technological efficiency and organizational readiness while paying limited attention to psychological trust as a determinant of AI substitution perceptions (Zhang et al., 2023). Most previous studies examine AI adoption from operational or technological perspectives without adequately exploring how emotional and psychological confidence influence acceptance behavior among accounting professionals. This represents a significant research gap because AI integration within accounting depends not only on technological capability but also on users' psychological willingness to rely on intelligent systems.

The present study addresses this gap by examining psychological trust as a critical predictor of perceptions regarding AI substitution in accounting frameworks. The study argues that accounting professionals who exhibit higher levels of trust toward AI technologies are more likely to perceive AI systems as having the potential to substitute repetitive and analytical accounting tasks. Trust reduces uncertainty, strengthens technological confidence, and enhances willingness to collaborate with AI-enabled accounting systems. Based on the above theoretical and empirical arguments, the following hypothesis is proposed:

H1: Psychological trust positively influences accounting professionals' perceptions regarding AI's potential to substitute selected accounting tasks.

#### 2.2.2 AI anxiety and AI substitution perception

AI anxiety refers to the feelings of fear, uncertainty, discomfort, and psychological stress individuals experience when interacting with or anticipating the growing influence of artificial intelligence technologies in professional environments (Wang and Wang, 2019). The rapid advancement of AI-enabled systems across industries has generated increasing concern regarding job displacement, technological dependency, skill obsolescence, and reduced human control over decision-making processes (Shi et al., 2024). In the accounting profession, these concerns have become particularly significant because AI technologies are increasingly perceived as capable of automating routine accounting tasks such as bookkeeping, auditing, taxation, financial reporting, and predictive analysis (Kokina and Davenport, 2017; Tu et al., 2020).

The integration of AI into accounting systems has contributed to perceptions that traditional accounting roles may change as repetitive and rule-based functions become increasingly supported by intelligent automation technologies. While AI is often associated with improved operational efficiency and analytical precision, many accounting professionals perceive these technologies as threats to their professional stability and future employability. The belief that AI may eventually replace certain accounting functions creates psychological resistance toward intelligent accounting systems. Such anxiety may reduce professionals' willingness to support AI integration and negatively influence perceptions regarding potential AI substitution in accounting tasks.

The Technology Acceptance Model (TAM) explains that emotional responses toward technology significantly influence adoption behavior (Davis, 1989). Individuals experiencing anxiety toward technological systems often perceive those systems as difficult, threatening, or risky, thereby reducing their willingness to adopt them. AI anxiety may therefore decrease accountants' perceptions of AI usefulness and acceptance within financial environments. Professionals who fear technological replacement are less likely to trust AI systems or perceive them as beneficial collaborators within accounting processes.

Similarly, Trust Theory suggests that uncertainty and fear weaken individuals' confidence in technological systems (Mayer et al., 1995). When accounting professionals perceive AI as unpredictable or as having the potential to substitute aspects of human expertise, they may develop resistance toward AI-enabled accounting frameworks. Anxiety regarding loss of professional autonomy, ethical concerns, and algorithmic decision-making may further intensify negative perceptions toward AI substitution.

Previous empirical studies support the negative relationship between technological anxiety and AI adoption behavior. (Li and Huang 2020) found that employees experiencing higher AI anxiety demonstrated lower willingness to adopt intelligent systems within organizational settings. Likewise, (Kelly et al. 2023) reported that fear regarding job displacement significantly reduces acceptance of AI technologies among professionals. In accounting environments, automation anxiety has emerged as a major psychological barrier affecting the successful implementation of intelligent accounting systems (Moll and Yigitbasioglu, 2019).

Furthermore, AI anxiety may be particularly prominent in the Chinese accounting sector due to the rapid pace of digital transformation and organizational automation. China's increasing investment in intelligent financial infrastructures has strengthened perceptions that some manual accounting functions may become increasingly automated. Consequently, accounting professionals may experience heightened uncertainty regarding future career opportunities, changing skill requirements, and technological expectations. Employees who perceive AI as a threat to their professional identity may become resistant toward AI-enabled accounting transformation.

The literature also suggests that anxiety negatively affects learning motivation and adaptability toward technological innovation. Professionals experiencing AI anxiety may avoid engaging with AI systems, resist technological training, and hesitate to integrate intelligent tools into accounting practices. Such resistance limits the development of positive perceptions regarding AI substitution within accounting frameworks. Although AI technologies are frequently viewed as capable of automating repetitive accounting activities, professionals experiencing high levels of AI anxiety are less likely to perceive AI as an acceptable substitute for human accountants. The governance implications of AI-enabled accounting transformation may also intensify anxiety among accounting professionals. As intelligent systems increasingly participate in financial control, compliance monitoring, and risk management activities, professionals may experience uncertainty regarding future responsibilities, accountability arrangements, and occupational relevance. (Bonelli 2026) suggests that transitions toward intelligent accounting systems frequently generate concerns regarding changing professional roles, redistribution of expertise, and the evolving relationship between human judgment and algorithmic decision-making. Such concerns may contribute to stronger perceptions of technological threat and lower acceptance of AI-enabled substitution.

Therefore, AI anxiety is expected to negatively influence perceptions regarding AI substitution in future accounting environments. AI anxiety may also reflect perceived job insecurity and threats to professional identity. Accounting professionals who view AI as undermining the distinctiveness and value of their expertise may exhibit stronger resistance toward perceived AI substitution. Recent workplace psychology research suggests that AI anxiety extends beyond fear of technological complexity to encompass concerns regarding employability, changing skill requirements, diminished professional relevance, and uncertainty surrounding future career trajectories in increasingly AI-enabled workplaces (Kelly et al., 2023; Budhwar et al., 2023). Such concerns may be particularly salient in accounting professions experiencing accelerated automation and digital transformation. Thus, the following hypothesis has been developed:

H2: AI anxiety negatively influences perceptions regarding AI's potential to substitute accounting tasks.

#### 2.2.3 Cognitive adaptability and AI substitution perception

Cognitive adaptability refers to an individual's capability to adjust cognitive strategies, thinking patterns, and problem-solving approaches in response to changing technological and environmental conditions (Haynie et al., 2010). In the context of artificial intelligence (AI)-enabled accounting systems, cognitive adaptability plays a critical role in determining how accounting professionals respond to technological transformation and whether they perceive AI as capable of substituting human accounting tasks. As accounting environments become increasingly digitized and data-driven, professionals are required to continuously learn, interpret, and integrate intelligent technologies into financial operations.

The accounting profession is currently undergoing substantial transformation due to the integration of AI technologies such as robotic process automation, machine learning, intelligent auditing systems, predictive analytics, and automated financial reporting. These intelligent systems are often perceived as capable of performing repetitive and analytical accounting functions more efficiently than traditional manual methods. However, professionals' perceptions regarding AI's role in accounting depend largely on their willingness and capability to adapt cognitively to these technological changes (Kokina and Davenport, 2017). Individuals with high cognitive adaptability are generally more open to innovation, flexible in learning new technologies, and capable of modifying existing work strategies according to technological requirements. Such individuals are better equipped to manage uncertainty and complexity associated with AI-driven accounting systems. They are also more likely to perceive AI technologies as opportunities for improving professional efficiency rather than threats to career stability. In contrast, professionals with lower adaptability may resist technological transformation due to difficulties in adjusting to changing accounting practices and digital work environments.

The Technology Acceptance Model (TAM) supports the argument that adaptable individuals are more likely to perceive technological systems as useful and easier to use (Davis, 1989). Cognitive adaptability reduces technological learning barriers and enhances users' confidence in engaging with AI systems. Adaptable professionals can understand AI functionalities more effectively, interpret AI-generated outputs accurately, and collaborate efficiently with intelligent systems (Peng et al., 2025). Consequently, they are more likely to accept the perceived substitution of repetitive accounting tasks by AI technologies. Furthermore, cognitive adaptability is particularly important in accounting because modern accounting functions increasingly require strategic interpretation of AI-generated financial insights. Accountants who possess adaptive cognitive capabilities can combine human judgment with intelligent technologies to improve decision-making quality. Such professionals are more capable of transitioning from traditional transactional roles toward analytical and strategic accounting functions in AI-enabled environments (Moll and Yigitbasioglu, 2019).

Previous studies indicate that cognitive adaptability positively influences innovation acceptance and digital transformation readiness. (Haynie et al. 2010) argue that cognitively adaptable individuals demonstrate greater flexibility in responding to uncertain and evolving environments. Similarly, (Armstrong and Mahmud 2008) found that adaptable individuals are more likely to embrace technological innovation and develop competencies necessary for changing professional environments. In financial and technological contexts, (Park and Woo 2022) reported that cognitive adaptability significantly enhances AI adoption behavior and technological collaboration among professionals.

Within the Chinese accounting sector, rapid digitalization and government-supported AI initiatives have intensified the need for professionals to adapt to intelligent accounting systems. Chinese enterprises increasingly rely on AI-enabled financial technologies to improve operational efficiency, support routine accounting tasks, and strengthen predictive financial analysis (Li et al., 2025). Consequently, accounting professionals who demonstrate higher levels of cognitive adaptability are more likely to perceive AI systems as effective substitutes for repetitive and analytical accounting functions. Moreover, cognitive adaptability enables professionals to manage technological uncertainty more effectively. AI systems frequently evolve through algorithmic learning and continuous system updates, requiring accountants to regularly acquire new technical competencies and modify work procedures. Adaptable individuals are more comfortable with continuous learning processes and are therefore more likely to support AI integration within accounting frameworks. Their ability to adjust to technological disruptions strengthens positive perceptions regarding the role of AI in future accounting systems.

Additionally, cognitive adaptability contributes to reduced resistance toward perceived AI substitution because adaptable professionals are less likely to perceive technological change as threatening. Instead, they are more likely to recognize the complementary relationship between human expertise and intelligent technologies. This perspective increases their willingness to accept AI-driven transformation within accounting professions. Based on these arguments, cognitive adaptability is expected to positively influence perceptions regarding AI substitution of human accounting tasks.

H3: Cognitive adaptability positively influences perceptions regarding AI's potential to substitute accounting tasks.

#### 2.2.4 Psychological trust and cognitive adaptability

Psychological trust plays a critical role in shaping individuals' adaptability toward technological transformation, particularly within AI-enabled professional environments. Trust refers to the willingness of individuals to rely on technological systems based on perceptions of competence, reliability, predictability, and integrity (Mayer et al., 1995). In accounting contexts, where AI systems increasingly perform complex financial tasks such as automated auditing, fraud detection, predictive analytics, and intelligent reporting, professionals must develop confidence in AI technologies before they can effectively adapt to technologically evolving work environments. The integration of AI into accounting frameworks requires accounting professionals to continuously modify work routines, acquire new digital competencies, and adjust cognitive strategies to accommodate intelligent systems. Such adaptation processes are strongly influenced by the level of trust individuals place in AI technologies. When accounting professionals perceive AI systems as trustworthy, transparent, and reliable, they become more willing to engage with technological learning processes and embrace organizational digital transformation. Trust reduces uncertainty and psychological resistance, thereby encouraging professionals to explore and adapt to AI-enabled accounting systems (Glikson and Woolley, 2020).

From the perspective of Trust Theory, trust functions as a psychological mechanism that facilitates cooperation between humans and intelligent systems. Individuals who trust AI technologies are more likely to perceive technological change as beneficial rather than threatening. Consequently, trusted systems generate feelings of confidence and security that support learning behavior and cognitive flexibility. In contrast, low levels of trust may increase skepticism and fear, reducing professionals' willingness to adapt to new technological environments (Gefen et al., 2003).

The Technology Acceptance Model (TAM) further supports the relationship between trust and cognitive adaptability. TAM suggests that technologies perceived as useful and reliable positively influence users' acceptance and engagement behaviors (Davis, 1989). When professionals trust AI systems, they are more likely to perceive these technologies as beneficial for improving accounting efficiency and performance. Such positive perceptions encourage individuals to invest cognitive effort in learning and integrating AI technologies into professional activities. Therefore, trust indirectly enhances adaptability by motivating users to interact positively with intelligent systems. In accounting environments, cognitive adaptability refers to professionals' ability to modify thinking patterns, learning approaches, and problem-solving strategies in response to technological transformation (Haynie et al., 2010). AI-driven accounting systems continuously evolve through machine learning and predictive algorithms, requiring accountants to remain flexible and technologically responsive. Professionals who trust AI systems are generally more open to experimentation, technological learning, and workflow modification, all of which contribute to higher cognitive adaptability.

Previous studies provide empirical support for the positive relationship between trust and adaptability in technological settings. (Gefen et al. 2003) found that trust significantly improves users' willingness to engage with digital systems and reduces uncertainty associated with technological complexity. Similarly, (Jöhnk et al. 2021) argued that trust in AI systems enhances organizational readiness for technological transformation by promoting learning orientation and behavioral flexibility. In financial and accounting contexts, professionals who trust intelligent technologies demonstrate greater acceptance of AI-enabled auditing tools, financial analytics platforms, and automated reporting systems (Issa et al., 2016).

Within the Chinese accounting industry, the rapid implementation of intelligent financial systems has increased the need for professionals to adapt cognitively to AI-driven environments. Organizations are increasingly adopting AI technologies to enhance efficiency, reduce operational costs, and improve decision-making capabilities. Accounting professionals who trust these intelligent systems are more likely to develop adaptive cognitive behaviors necessary for successful collaboration with AI technologies. Trust enables professionals to view AI not as a threat but as a strategic tool supporting accounting innovation and professional development. Furthermore, psychological trust may reduce the cognitive burden associated with technological uncertainty. Professionals who trust AI systems experience lower levels of technological fear and resistance, allowing them to focus on skill development and technological learning. This enhances their ability to adapt to changing accounting environments and strengthens readiness for AI-enabled transformation. Therefore, psychological trust is expected to positively influence cognitive adaptability among accounting professionals in AI-integrated accounting environments.

H4: Psychological trust positively influences cognitive adaptability.

#### 2.2.5 AI anxiety and cognitive adaptability

Artificial intelligence (AI) anxiety has emerged as a significant psychological challenge influencing employees' adaptation to technologically advanced work environments. AI anxiety refers to feelings of fear, uncertainty, discomfort, and psychological stress associated with the increasing use of intelligent technologies in professional settings (Wang and Wang, 2019). As AI systems become increasingly integrated into accounting operations, professionals may experience concerns regarding job displacement, technological dependency, skill obsolescence, and reduced professional relevance. These emotional responses can negatively affect individuals' willingness and ability to cognitively adapt to AI-enabled accounting systems. Cognitive adaptability refers to an individual's capability to modify cognitive strategies, thinking patterns, and learning approaches in response to changing technological and environmental conditions (Haynie et al., 2010). In the context of accounting, cognitive adaptability enables professionals to understand intelligent systems, learn emerging digital tools, and integrate AI technologies into accounting practices effectively. Adaptable individuals are generally more open to innovation and technological transformation because they possess greater flexibility in adjusting to changing work environments.

However, AI anxiety may weaken cognitive adaptability by creating psychological resistance toward technological learning and experimentation. Individuals experiencing fear and uncertainty regarding AI systems are less likely to engage positively with technological changes. Anxiety often reduces confidence, increases stress, and limits willingness to acquire new technological competencies. In accounting environments, professionals who perceive AI as a threat to career stability may avoid interacting with intelligent systems and resist organizational digital transformation initiatives.

The Technology Acceptance Model (TAM) suggests that negative emotional reactions toward technology reduce users' willingness to engage with and adapt to technological systems (Davis, 1989). Anxiety increases perceptions regarding technological complexity and uncertainty, thereby reducing individuals' confidence in learning and using AI systems effectively. Similarly, psychological theories of stress and adaptation indicate that fear-based perceptions negatively influence cognitive flexibility and problem-solving behavior (Lazarus and Folkman, 1984). When professionals perceive technological change as threatening rather than beneficial, they become less adaptable to evolving work environments.

Previous studies support the negative relationship between technological anxiety and cognitive adaptability. (Thatcher and Perrewé 2002) found that technological anxiety significantly reduces learning confidence and limits individuals' ability to adjust to new technological systems. Likewise, (Li and Huang 2020) reported that AI anxiety negatively influences employees' willingness to develop AI-related competencies and engage with intelligent technologies. In organizational settings, employees experiencing higher levels of AI anxiety are more likely to resist technological innovation and exhibit lower adaptability toward digital transformation processes. Within accounting professions, growing perceptions that bookkeeping, auditing, taxation, and financial reporting functions may become increasingly automated have intensified concerns regarding the future role of human accountants (He et al., 2025). Professionals who fear replacement by intelligent systems may perceive AI technologies as disruptive and threatening, thereby reducing their motivation to adapt cognitively to AI-driven accounting environments. Furthermore, anxiety regarding technological complexity may discourage accountants from participating in AI-related training programs and technological skill development initiatives.

In China, the accounting industry is undergoing rapid digital transformation driven by governmental AI initiatives and organizational investments in intelligent financial systems. As accounting firms increasingly implement AI-enabled technologies, professionals are required to continuously update their skills and adapt to changing technological expectations. However, individuals experiencing high AI anxiety may struggle to cognitively adjust to these technological transitions, thereby limiting successful AI integration within accounting frameworks. Therefore, based on existing theoretical and empirical evidence, AI anxiety is expected to negatively influence cognitive adaptability among accounting professionals.

H5: AI anxiety negatively influences cognitive adaptability.

#### 2.2.6 Mediating role of cognitive adaptability

Cognitive adaptability plays a crucial mediating role in explaining how psychological trust influences perceptions regarding artificial intelligence (AI) substitution within future accounting frameworks. Cognitive adaptability refers to an individual's capability to modify cognitive strategies, learning patterns, and problem-solving approaches in response to changing technological environments (Haynie et al., 2010). In rapidly evolving AI-driven accounting systems, accounting professionals are required to continuously adapt to intelligent technologies, automated financial platforms, predictive analytics, and machine-learning applications. Therefore, cognitive adaptability becomes an essential psychological mechanism facilitating technological transition and AI acceptance. The relationship between psychological trust and AI substitution perception may not occur directly in all circumstances. Instead, trust in AI systems may first enhance individuals' willingness to cognitively engage with technological transformation, which subsequently increases acceptance of AI-enabled accounting systems. Professionals who trust AI technologies are more likely to perceive intelligent systems as reliable, beneficial, and worthy of exploration. This positive perception encourages them to acquire new technological competencies, modify existing accounting practices, and develop adaptive cognitive strategies necessary for working effectively with AI systems.

Trust Theory suggests that individuals are more willing to engage with uncertain technological systems when they perceive those systems as trustworthy, competent, and dependable (Mayer et al., 1995). Trust reduces psychological uncertainty and fear associated with technological change, thereby creating a supportive environment for learning and adaptation. In accounting contexts, professionals who trust AI-generated financial outputs are more likely to invest effort in understanding intelligent systems and integrating them into accounting workflows. Such trust-driven engagement enhances cognitive flexibility and adaptability toward AI-enabled accounting transformation.

Similarly, the Technology Acceptance Model (TAM) explains that positive perceptions regarding technology increase users' willingness to interact with and adopt technological systems (Davis, 1989). Cognitive adaptability strengthens this process by enabling individuals to translate favorable technological perceptions into adaptive behavior. Professionals possessing strong adaptability are more capable of adjusting to AI-driven accounting systems, learning new digital skills, and responding positively to technological disruption. Consequently, cognitive adaptability acts as a psychological bridge connecting trust in AI systems with acceptance of perceived AI substitution in accounting functions.

In accounting environments, adaptable professionals are generally more open to integrating AI into financial reporting, auditing, fraud detection, taxation, and predictive analytics. They perceive technological transformation as an opportunity for professional development rather than a threat to occupational relevance. Conversely, professionals with lower adaptability may struggle to translate trust into effective AI engagement because they lack the cognitive flexibility required to adjust to technologically advanced accounting environments.

Prior empirical studies support the mediating role of adaptability in technology adoption contexts. (Haynie et al. 2010) argued that cognitive adaptability enables individuals to respond effectively to uncertain and dynamic environments by adjusting cognitive processes according to situational demands. Likewise, (Armstrong and Mahmud 2008) found that adaptable individuals are more capable of integrating innovative technologies into professional activities. In AI-related contexts, adaptable professionals demonstrate greater willingness to collaborate with intelligent systems and accept technological transformation (Jiang et al., 2012; Park and Woo, 2022).

Within the Chinese accounting sector, the increasing implementation of AI-enabled accounting systems requires professionals to continuously develop technological competencies and adaptive learning behaviors (Li et al., 2024). Accounting professionals who trust intelligent systems are more likely to develop cognitive adaptability by engaging in AI-related training, experimenting with intelligent financial tools, and restructuring traditional accounting practices. This adaptability subsequently increases their perceptions regarding AI's potential to substitute repetitive and analytical accounting tasks. Furthermore, cognitive adaptability helps reduce psychological resistance toward AI-driven transformation. Professionals capable of adjusting cognitive strategies are better able to manage uncertainties associated with automation, technological complexity, and changing workplace requirements. As a result, they are more likely to perceive AI systems as collaborative tools that may support accounting efficiency and productivity.

The mediating role of cognitive adaptability is therefore theoretically and practically significant because it explains the psychological mechanism through which trust influences AI substitution perceptions. Trust alone may not be sufficient to encourage AI acceptance unless individuals possess the cognitive capability to adapt to intelligent technologies. Cognitive adaptability transforms psychological trust into proactive technological engagement, thereby strengthening acceptance of AI-enabled accounting systems. Based on these theoretical arguments and empirical findings, the present study proposes the following hypothesis:

H6: Cognitive adaptability mediates the relationship between psychological trust and perceptions regarding AI's potential to substitute accounting tasks.

**Figure 2:**
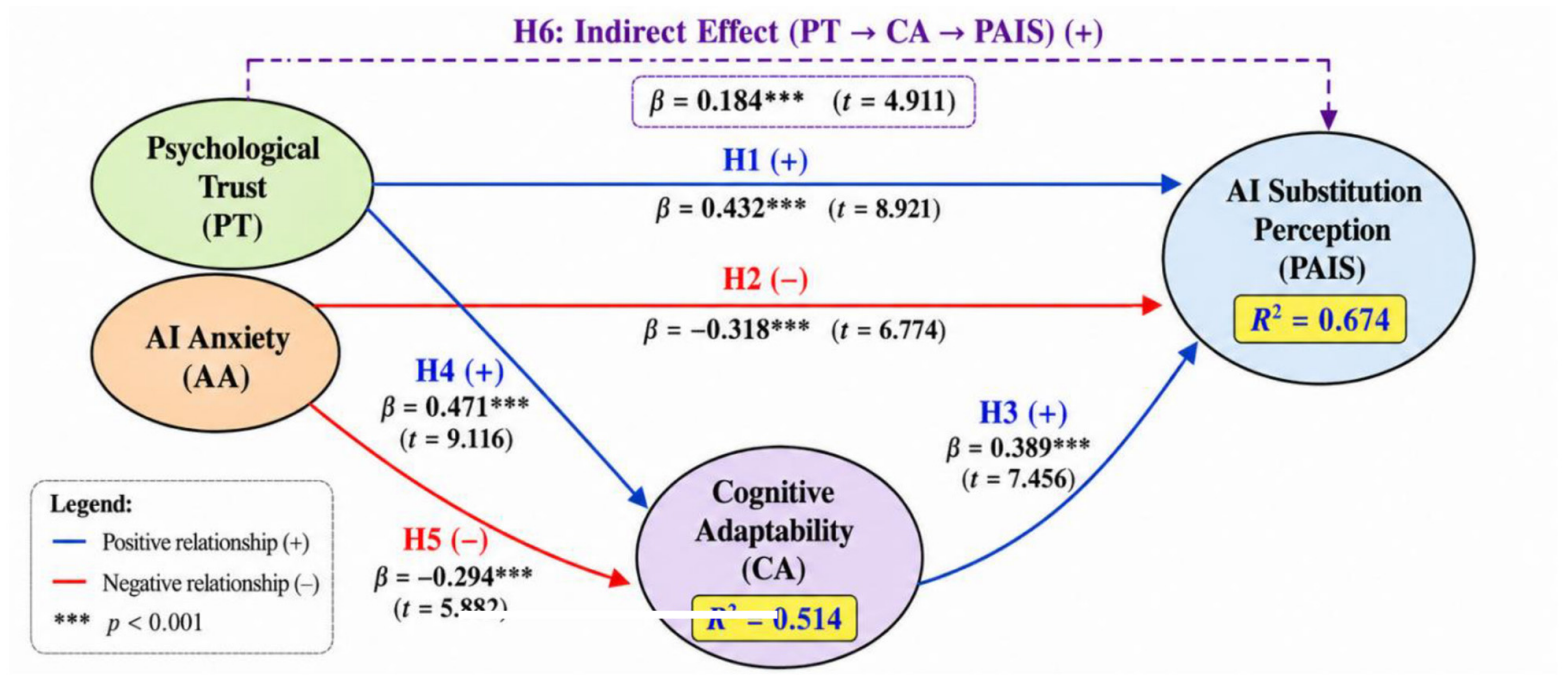
Structural model linking hypotheses, path coefficients, and directions (PLS-SEM Results). Values shown are standardized path coefficients (β) with *t*-values in parentheses. Blue color indicates a positive relationship (+); red color indicates a negative relationship (-). *** indicates significance at *p* < 0.001. H6 represents the indirect (mediated) effect of Psychological Trust on AI Substitution Perception through Cognitive Adaptability.

Figure 2 presents the proposed structural model illustrating the hypothesized relationships among psychological trust, AI anxiety, cognitive adaptability, and perceived AI substitution within future accounting frameworks. The figure displays the standardized path coefficients (β), directional signs, and significance levels obtained from the PLS-SEM analysis. Positive relationships are indicated by positive path coefficients, demonstrating that psychological trust and cognitive adaptability increase accounting professionals' perceptions regarding AI's potential to substitute selected accounting tasks. In contrast, negative path coefficients indicate that AI anxiety reduces both cognitive adaptability and perceptions of AI substitution. The figure also depicts the mediating role of cognitive adaptability in the relationship between psychological trust and perceived AI substitution, highlighting the psychological mechanism through which trust enhances acceptance of AI-enabled transformation. Furthermore, the reported *R*^2^ values indicate the proportion of variance explained by the model, suggesting satisfactory explanatory power in predicting cognitive adaptability and perceived AI substitution among accounting professionals.

## 3 Methodology

### 3.1 Research design

The present study adopted an explanatory sequential mixed methods design characterized by a quantitative-dominant orientation followed by a supplementary qualitative phase. This design was considered appropriate because the study sought not only to test theoretically derived relationships among psychological trust, AI anxiety, cognitive adaptability, and perceived AI substitution, but also to understand the underlying psychological mechanisms through which accounting professionals interpret AI-enabled transformation. The study supports a perception-based design in which all constructs are measured through self-reported evaluations. Consequently, the findings reflect respondents' beliefs and expectations regarding AI substitution and cannot be interpreted as evidence of AI's actual capability, accounting performance, or occupational replacement. The quantitative component constituted the primary phase of the investigation and reflected a predominantly positivist orientation. Positivism assumes that social and psychological phenomena can be objectively measured, empirically observed, and statistically examined through systematic procedures. Consistent with this perspective, the study employed structured questionnaires and Partial Least Squares Structural Equation Modeling (PLS-SEM) to test the hypothesized causal relationships derived from TAM, Trust Theory, and contemporary psychological perspectives on human–AI interaction. The quantitative phase enabled the identification of significant associations, explanatory power, and mediating effects among the proposed constructs.

However, organizational psychology phenomena are rarely fully understood through objective measurement alone. Employees' responses to technological change are embedded within subjective experiences involving meaning construction, professional identity, emotional reactions, and contextual interpretations (Zhu et al., 2025). Accounting professionals may exhibit similar levels of trust or anxiety for different underlying reasons shaped by their organizational experiences, career trajectories, and perceptions of occupational change. Therefore, an exclusively positivist approach would have been insufficient to explain why these psychological relationships emerged and how participants interpreted AI-enabled transformation within their professional environments. Accordingly, the study incorporated an interpretivist qualitative phase to complement and deepen the quantitative findings. Interpretivism recognizes that individuals actively construct meanings based on their social and professional contexts and that understanding these meanings is essential when investigating complex psychological phenomena. Semi-structured interviews with accounting professionals were therefore conducted after completion of the quantitative analysis to explore how participants understood trust in AI systems, experienced AI-related anxiety, adapted cognitively to technological change, and negotiated the evolving boundaries between human expertise and intelligent systems.

The adoption of both positivist and interpretivist assumptions reflects the interdisciplinary nature of organizational psychology research (Jackson et al., 2006). Organizational psychology simultaneously examines objectively measurable psychological constructs and the subjective processes through which individuals make sense of workplace experiences. Constructs such as trust, anxiety, and adaptability possess quantifiable dimensions that permit hypothesis testing, while their manifestation within organizational settings is shaped by interpretation, identity, social interaction, and contextual meaning. Consequently, combining these philosophical perspectives provides a more comprehensive understanding of how accounting professionals psychologically respond to AI-enabled occupational transformation. This study therefore implemented a quantitative-dominant explanatory sequential mixed methods design in which the qualitative phase served an explanatory rather than equal-status function. The findings from the quantitative phase informed participant selection and guided the development of interview questions, enabling the qualitative inquiry to focus specifically on explaining, elaborating, and contextualizing statistically significant results. Through this process, the study moved beyond determining whether relationships existed to understanding why such relationships emerged.

Furthermore, the integration of quantitative and qualitative findings involved result triangulation and the development of meta-inferences. Result triangulation enabled the convergence, complementarity, and comparison of evidence obtained through both methodological strands, thereby strengthening the credibility and validity of the conclusions. Quantitative findings identified the direction and magnitude of psychological relationships, whereas qualitative evidence provided richer explanations of the mechanisms underlying those relationships. The integration of these findings generated comprehensive meta-inferences regarding how psychological trust, AI anxiety, and cognitive adaptability shape accounting professionals' perceptions of AI substitution within future accounting frameworks. Accordingly, the explanatory sequential mixed methods approach employed in this study was not merely a procedural combination of methods but a theoretically and philosophically justified strategy aligned with the characteristics of organizational psychology. The design enabled rigorous hypothesis testing while preserving sensitivity to the subjective experiences through which professionals interpret and adapt to technologically mediated workplace transformation.

**Figure 3:**
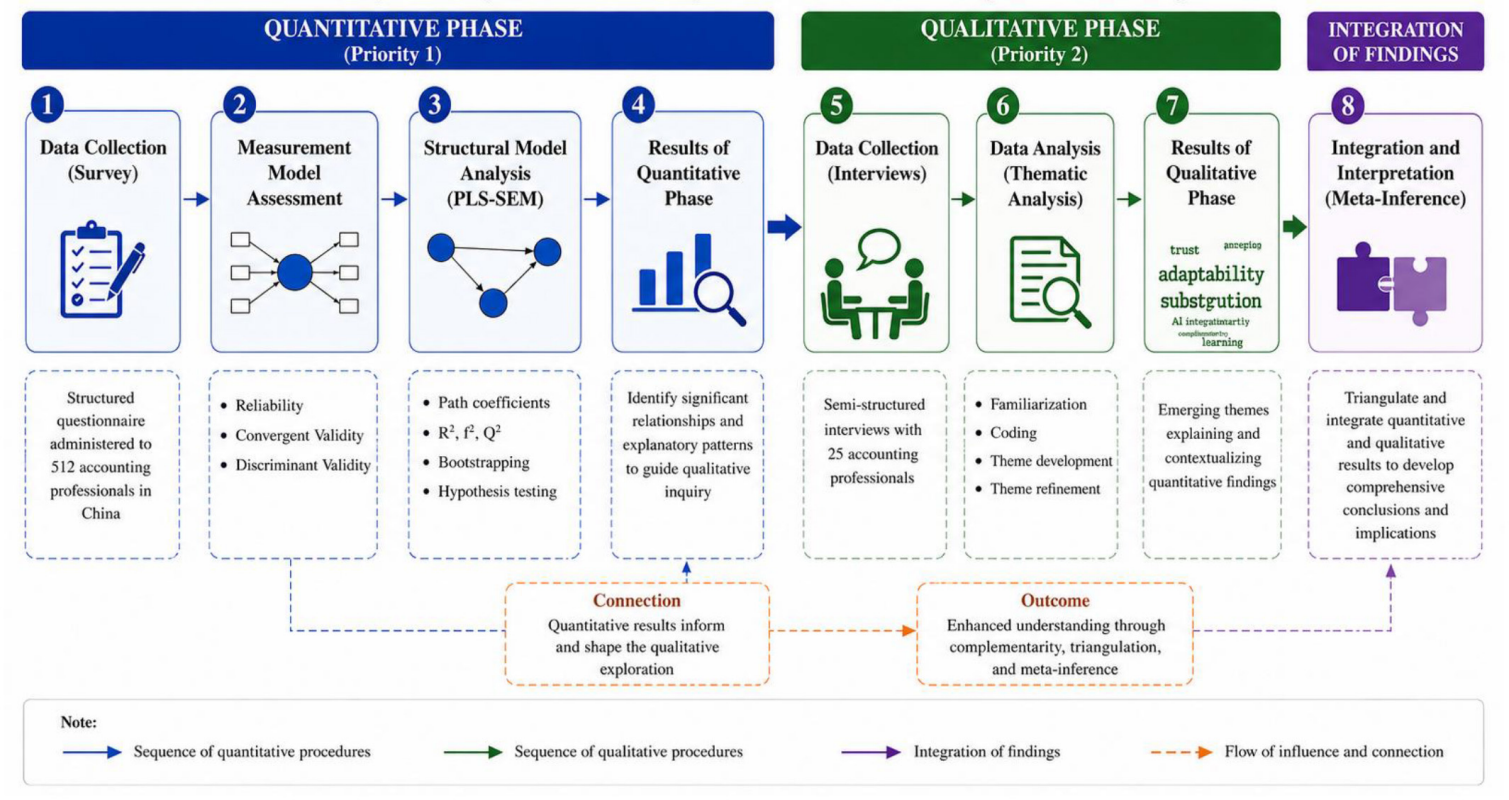
Explanatory sequential mixed-method research design. The quantitative phase was conducted first to test the hypothesized model using PLS-SEM. The qualitative phase followed to explain and contextualize the quantitative findings.

Figure 3 illustrates the explanatory sequential mixed-method research design employed in this study. The quantitative phase was conducted first to test the hypothesized relationships using PLS-SEM based on survey data from 512 accounting professionals. The findings from this phase informed the subsequent qualitative phase involving semi-structured interviews with 25 participants to provide deeper explanations and contextual understanding of the statistical results. Finally, the integration of quantitative and qualitative findings enabled triangulation and the development of comprehensive meta-inferences regarding how psychological factors shape perceptions of AI substitution in future accounting frameworks.

### 3.2 Research context

China was selected as the research context because of its rapid advancement in artificial intelligence adoption and digital financial transformation. The Chinese government has heavily invested in intelligent technologies through national digitalization strategies and AI development initiatives, encouraging organizations to integrate AI-enabled systems across industries (Wang et al., 2025). Chinese accounting firms and corporations increasingly utilize intelligent financial systems for bookkeeping, auditing, taxation, financial forecasting, fraud detection, and risk management. This rapid integration of AI technologies makes China an appropriate context for examining whether AI can serve as a substitute for human accounting tasks in future accounting frameworks.

Furthermore, the Chinese accounting sector is currently experiencing substantial technological transformation, creating both opportunities and psychological challenges for accounting professionals (Li et al., 2026). The increasing implementation of AI-driven systems has generated concerns regarding job displacement, professional relevance, technological uncertainty, and skill adaptation. Therefore, examining the psychological dimensions of AI acceptance within the Chinese accounting environment provides significant practical and theoretical relevance.

### 3.3 Target population and sampling

The target population of the study consisted of accounting professionals encompassing auditors, tax consultants, finance managers, accounting academics, and financial analysts employed in Chinese public and private organizations. Respondents were selected because they possessed direct experience and exposure to accounting technologies and AI-enabled financial systems. The study utilized a non-probability purposive sampling technique because respondents were selected based on their professional knowledge and familiarity with AI applications in accounting. Purposive sampling was considered appropriate because the study specifically required participants who had experience working within digitally transforming accounting environments. Respondents were recruited from major Chinese economic and technological centers, including Beijing, Shanghai, Shenzhen, Guangzhou, and Hangzhou, where AI adoption in accounting systems is highly prevalent. Majority of respondents were recruited from China's first-tier cities where digital infrastructure, AI investment, and the implementation of intelligent accounting systems are relatively advanced. Consequently, the participants were more likely to possess direct exposure to AI-enabled accounting technologies and organizational digital transformation initiatives. While this sampling strategy enhanced the relevance of the study for examining psychological responses within technologically mature environments, it may limit the representativeness of accounting professionals working in regions characterized by lower levels of digital development and technological readiness.

To ensure the suitability of respondents for the study objectives, a screening procedure was implemented prior to data collection. Participants were required to satisfy two eligibility criteria. First, they had to be currently employed in an accounting-related role, including auditing, taxation, financial management, financial analysis, or accounting education, within either public or private organizations in China. Second, respondents were required to report direct exposure to AI-enabled accounting technologies through system usage, supervisory responsibilities, organizational implementation activities, or participation in AI-related training initiatives. Examples of such technologies included robotic process automation, intelligent auditing tools, predictive analytics platforms, automated financial reporting systems, and AI-supported decision-support applications. These criteria ensured that respondents possessed adequate professional knowledge and practical familiarity to provide informed evaluations regarding the perceived substitutive potential of AI in accounting functions.

Professional status was verified using multiple approaches depending on the recruitment channel. Participants recruited through professional accounting associations were requested to indicate their professional membership status and current occupational designation. For respondents recruited through LinkedIn professional groups, publicly available occupational information contained within professional profiles was used to confirm role suitability. Individuals accessed through university networks and organizational contacts were asked to provide information regarding their current position, employing organization, years of professional experience, and primary accounting responsibilities. Responses exhibiting incomplete occupational information, inconsistencies across screening questions, or indications that participants lacked relevant accounting experience were excluded from further analysis.

For the quantitative phase, 600 structured questionnaires were distributed electronically through professional accounting associations, LinkedIn professional groups, university networks, and organizational contacts. Before proceeding to the main questionnaire, participants completed screening questions designed to confirm their eligibility and assess their exposure to AI-enabled accounting systems. After eliminating incomplete responses, questionnaires with inconsistent professional information, duplicate submissions, and responses failing the eligibility criteria, a total of 512 valid questionnaires were retained for final analysis, yielding an effective response rate of 85.3 percent. The sample size exceeded the minimum threshold required for PLS-SEM analysis and provided sufficient statistical power for hypothesis testing (Hair et al., 2022).

The final sample represented a diverse cross-section of accounting professionals operating within technologically advanced regions of China. Participants were drawn from multiple accounting roles and organizational settings, thereby enhancing the breadth of perspectives captured in the study. The inclusion of respondents with demonstrable exposure to AI-enabled accounting environments strengthened the credibility of the findings and increased confidence that participants were well-positioned to evaluate how psychological trust, AI anxiety, and cognitive adaptability shape perceptions regarding AI's potential to substitute selected accounting tasks within future accounting frameworks.

Table 1 presents the demographic characteristics of the participants. The demographic analysis was undertaken to provide an overview of the characteristics of the respondents participating in the study and to assess whether the sample adequately represented accounting professionals with sufficient experience to evaluate the implications of AI-enabled transformation within accounting environments. A total of 512 valid responses were obtained from auditors, tax consultants, finance managers, accounting academics, and financial analysts employed across major Chinese cities.

**Table 1:** Demographic characteristics of respondents (*N* = 512).

Demographic variable	Category	Frequency (*n*)	Percentage (%)
Gender	Male	276	53.9
	Female	236	46.1
Age (years)	25–30	84	16.4
	31–35	118	23.0
	36–45	223	43.6
	Above 45	87	17.0
Educational qualification	Bachelor's degree	171	33.4
	Master's degree	296	57.8
	Doctoral degree	45	8.8
Professional role	Financial analyst	127	24.8
	Auditor	120	23.4
	Finance manager	110	21.5
	Tax consultant	86	16.8
	Accounting academics	69	13.5
Professional experience	Less than 5 years	98	19.1
	6–10 years	154	30.1
	11–15 years	128	25.0
	16–20 years	77	15.0
	More than 20 years	55	10.8

The findings as reported in Table 1 reveal a relatively balanced gender distribution, with male respondents constituting 53.9% of the sample and female respondents accounting for 46.1%. Regarding age, the majority of participants (66.6%) were between 31 and 45 years of age, suggesting that most respondents were in their mid-career stages and possessed substantial professional maturity. Respondents aged above 45 years represented 17.0% of the sample, while younger professionals aged 25–30 years accounted for 16.4%. In terms of educational attainment, the respondents were highly qualified, with 57.8% holding master's degrees and 8.8% possessing doctoral qualifications. This indicates that the sample largely comprised highly educated professionals capable of understanding the implications of emerging technologies in accounting practice. With respect to professional roles, financial analysts constituted the largest proportion of the sample (24.8%), followed by auditors (23.4%), finance managers (21.5%), tax consultants (16.8%), and accounting academics (13.5%). The diversity of professional backgrounds enhances the comprehensiveness of the findings by capturing perspectives from multiple domains of accounting practice. Professional experience further demonstrates the suitability of the sample for the study objectives. Approximately 80.9% of the respondents had more than 5 years of professional experience, indicating substantial exposure to technological developments and evolving accounting practices. The predominance of experienced professionals suggests that the respondents were well-positioned to provide informed evaluations regarding the perceived potential of AI to substitute selected accounting tasks within future accounting frameworks.

To further enhance the transparency of the sampling profile, the distribution of respondents across organizational settings, industries, and cities was examined. Approximately 31.4% of respondents were employed in private enterprises, 24.6% in public accounting firms, 18.8% in financial institutions, 14.3% in state-owned enterprises, and 10.9% in educational institutions. With respect to industrial sectors, respondents represented manufacturing (27.1%), financial services (24.4%), technology and telecommunications (19.7%), professional services (16.2%), and other sectors (12.6%). Geographically, the sample included participants from Beijing (21.7%), Shanghai (24.2%), Shenzhen (20.3%), Guangzhou (18.8%), and Hangzhou (15.0%). The diversity of organizational and regional representation strengthens the generalizability of the findings within technologically advanced accounting environments in China.

The qualitative phase involved 25 semi-structured interviews with senior accountants, auditors, finance executives and accounting technology specialists. Participants were selected using purposive and snowball sampling techniques to ensure that respondents possessed substantial experience regarding AI implementation within accounting systems. Interviewees were required to demonstrate direct experience with AI-enabled accounting applications or involvement in organizational digital transformation initiatives. Thematic saturation was achieved after the twenty-third interview, and two additional interviews were conducted to confirm the stability of emerging themes.

A semi-structured interview protocol was developed to explore accounting professionals' experiences and interpretations of AI-enabled transformation in greater depth. The interview guide consisted of broad, open-ended questions rather than hypothesis-driven prompts to encourage participants to reflect on their lived experiences. Questions focused on: (i) participants' experiences with AI technologies in accounting practice; (ii) perceived opportunities and challenges associated with AI integration; (iii) concerns regarding professional identity, job security, and future career trajectories; (iv) experiences of learning and adapting to AI-enabled environments; and (v) perceptions regarding the future relationship between human accountants and intelligent systems. Follow-up probes were used to clarify meanings and elicit concrete examples. The interview guide was reviewed by two accounting academics with expertise in qualitative methods to ensure content validity and clarity. The complete interview protocol is provided in Supplementary Appendix S1.

To enhance transparency and provide a richer understanding of the qualitative sample, Table 2 presents the professional characteristics of the 25 interview participants involved in the explanatory sequential mixed-method phase of the study. The interview participants were purposively selected because of their direct experience with AI-enabled accounting systems and their ability to provide in-depth reflections on the psychological and professional implications of AI integration within accounting practice. The sample was designed to capture diverse perspectives across different accounting functions, levels of professional seniority, and varying degrees of exposure to intelligent technologies.

**Table 2:** Profile of interview participants (*n* = 25).

Participant code	Professional role	Years of experience	City
P1	Auditor	17	Shanghai
P2	Finance manager	14	Beijing
P3	Tax consultant	16	Guangzhou
P4	Accounting academic	19	Shenzhen
P5	Financial analyst	9	Hangzhou
P6	Senior accountant	12	Beijing
P7	Auditor	11	Shanghai
P8	Finance executive	21	Shenzhen
P9	Tax consultant	16	Guangzhou
P10	Financial analyst	10	Hangzhou
P11	Accounting academic	18	Beijing
P12	Accounting technology specialist	11	Shenzhen
P13	Senior accountant	14	Shanghai
P14	Auditor	20	Beijing
P15	Finance manager	16	Guangzhou
P16	Financial analyst	8	Shenzhen
P17	Tax consultant	12	Hangzhou
P18	Accounting technology specialist	10	Shanghai
P19	Accounting academic	24	Beijing
P20	Senior accountant	12	Guangzhou
P21	Auditor	18	Shenzhen
P22	Finance manager	13	Hangzhou
P23	Financial analyst	9	Beijing
P24	Tax consultant	17	Shanghai
P25	Senior accountant	12	Shenzhen

The participants represented a broad cross-section of the Chinese accounting profession, including auditors, finance managers, tax consultants, financial analysts, accounting academics, accounting technology specialists, and senior accountants from major economic centers such as Beijing, Shanghai, Shenzhen, Guangzhou, and Hangzhou. Their professional experience ranged from 8 to 24 years, ensuring that participants possessed sufficient occupational maturity to comment on both traditional accounting practices and emerging AI-driven transformations. Additionally, participants varied in their level of exposure to AI systems, ranging from moderate use of intelligent accounting tools to extensive involvement in AI-supported financial decision-making processes. This heterogeneity enhanced the credibility and transferability of the qualitative findings by enabling the exploration of multiple viewpoints regarding AI substitution, occupational adaptation, and the future of accounting work.

### 3.4 Data collection procedure

Primary data were collected between November 2025 and March 2026 using an explanatory sequential mixed-method design. The quantitative phase was conducted first between November 2025 and January 2026 through the administration of structured questionnaires to accounting professionals across major Chinese cities. Before accessing the questionnaire, participants were provided with an electronic participant information sheet explaining the purpose of the study, voluntary nature of participation, confidentiality safeguards, and their right to withdraw without consequence. Electronic informed consent was obtained prior to questionnaire completion. To ensure linguistic equivalence and cultural appropriateness, the questionnaire was initially developed in English and translated into Chinese using a back-translation procedure. A bilingual expert translated the instrument into Chinese, after which an independent bilingual translator, who was not involved in the initial translation, translated it back into English. Any discrepancies between the original and back-translated versions were discussed and resolved to ensure conceptual consistency. Prior to the main survey, a pilot study involving 40 participants representing different accounting job roles and age groups was conducted to assess the clarity, relevance, and comprehensibility of the questionnaire items. Based on participants' feedback, ambiguous items were revised and expressions were refined to improve readability and contextual suitability, thereby strengthening the content validity of the measurement instrument.

Following the quantitative analysis, the qualitative phase was undertaken between February and March 2026. Semi-structured interviews were conducted with 25 purposively selected accounting professionals to contextualize and explain the statistical findings. Prior to each interview, participants received detailed information regarding the study objectives and procedures and provided informed consent before participation. With participants' consent, interviews were audio-recorded and later transcribed for thematic analysis. All interview recordings were transcribed verbatim to preserve the accuracy and richness of participants' accounts. To enhance the credibility of the qualitative data and minimize manual transcription errors, two members of the research team independently cross-checked the transcripts against the original audio recordings. Any discrepancies were discussed and resolved through consensus before the commencement of thematic analysis. Subsequently, data cleaning, transcription verification, coding, and formal quantitative and qualitative analyses were completed in April 2026.

The qualitative phase involved semi-structured interviews conducted through online meeting platforms and face-to-face interactions depending on participants' availability. Each interview lasted approximately 40–60 min. The interviews focused on participants' experiences with AI-enabled accounting systems, perceptions regarding AI substitution, psychological trust, anxiety concerning automation, and adaptability toward technological changes.

### 3.5 Development and preliminary validation of the perceived AI substitution scale

The Perceived AI Substitution Scale (PAIS) was developed specifically for the present study because existing accounting and AI literature does not provide a validated instrument directly measuring professionals' perceptions regarding AI's potential substitution of accounting tasks. The scale was designed as an exploratory measure intended to capture subjective expectations concerning future AI involvement in accounting activities rather than actual technological capability or occupational replacement.

The scale-development process followed established recommendations for exploratory construct development (Churchill Jr, 1979; DeVellis, 2017; MacKenzie et al., 2011) and involved four stages:

Stage 1: Conceptual Domain Specification

The conceptual domain was defined through an extensive review of literature concerning AI adoption, intelligent automation, accounting digitalization, occupational change, algorithmic decision-making, and human–AI collaboration. The review indicated that discussions regarding AI substitution frequently involve perceptions that intelligent systems may perform routine, analytical, and information-processing tasks traditionally undertaken by professionals. Accordingly, PAIS was defined as:

“Accounting professionals' perceptions regarding the potential of AI systems to undertake selected accounting tasks within future accounting environments.”

This definition intentionally focuses on perceived potential rather than actual replacement.

Stage 2: Item Generation

Initial items were generated from themes repeatedly identified in prior literature discussing automation, AI-enabled accounting systems, intelligent auditing, and technological transformation of professional work. Particular attention was given to ensuring that items reflected perceived task substitution rather than general technology acceptance, trust, usefulness, or adoption intentions.

Stage 3: Expert Review and Content Validation

To establish content validity, the preliminary item pool was reviewed by a panel consisting of six experts, including three accounting academics specializing in digital accounting systems, two organizational psychology scholars with expertise in technology adoption, and one senior accounting practitioner experienced in AI-enabled financial systems.

Experts were asked to evaluate item clarity, conceptual relevance, comprehensiveness, and representativeness of the construct domain. Based on their recommendations, several items were revised to improve clarity and reduce conceptual overlap with technology acceptance and job insecurity constructs. Items deemed excessively broad or redundant were removed.

The expert review process supported the content validity of the scale by indicating that the retained items adequately represented perceptions regarding AI's potential involvement in accounting tasks while remaining conceptually distinct from trust, anxiety, and adaptability.

Stage 4: Pilot Testing

The revised instrument was pilot tested with 35 accounting professionals possessing experience with AI-enabled accounting technologies. Participants provided feedback regarding wording clarity, item relevance, and questionnaire comprehensibility. Minor linguistic modifications were subsequently introduced before full-scale data collection.

Because PAIS represents a newly developed measure, the present study treats it as an exploratory construct. Consequently, findings involving PAIS should be interpreted as preliminary evidence requiring further validation in future studies across different occupational, industrial, and cultural contexts.

### 3.6 Measurement of variables

The study measured all constructs using multi-item scales adopted and modified from established literature. A five-point Likert scale ranging from 1 = strongly disagree to 5 = strongly agree was utilized to measure respondents' perceptions.

The Table 3 presents constructs and their items with sources. Psychological trust was measured using items adapted from (Mayer et al. 1995) and (Glikson and Woolley 2020). The construct evaluated respondents' confidence regarding the reliability, transparency, accuracy, and competence of AI-enabled accounting systems. AI anxiety was measured using scales adapted from (Wang and Wang 2019). The items assessed feelings of uncertainty, fear, discomfort, technological insecurity, and concerns regarding job displacement caused by AI integration within accounting systems. Cognitive adaptability was measured using items adapted from (Haynie et al. 2010). The construct evaluated respondents' ability to modify cognitive strategies, adapt to technological changes, learn AI-related accounting tools, and integrate intelligent systems into professional activities. AI substitution perception was conceptualized as respondents' subjective beliefs regarding the extent to which AI could potentially perform selected accounting tasks in future accounting environments. AI substitution perception was measured using self-developed items based on existing AI-accounting literature. The construct assessed respondents' beliefs regarding AI's capability to substitute repetitive, analytical, and decision-support accounting tasks in future accounting frameworks. The construct does not capture actual AI performance, objective task completion accuracy, or verified replacement of professional judgment.

**Table 3:** Measurement items and sources.

Construct	Code	Measurement item	Source
Psychological trust (PT)	PT1	I believe that AI-enabled accounting systems produce reliable outputs	Mayer et al., 1995; Glikson and Woolley, 2020
	PT2	AI systems can accurately perform accounting functions without significant errors	Adapted from (Mayer et al. 1995); (Glikson and Woolley 2020)
	PT3	I trust the recommendations and outputs generated by AI-based accounting systems	Adapted from (Mayer et al. 1995); (Glikson and Woolley 2020)
	PT4	AI-enabled accounting systems are dependable in professional accounting settings	Adapted from (Mayer et al. 1995); (Glikson and Woolley 2020)
AI anxiety (AA)	AA1	I feel anxious about the increasing use of AI technologies in accounting	Wang and Wang, 2019
	AA2	I worry that AI may reduce the importance of accountants' professional roles	Adapted from (Wang and Wang 2019)
	AA3	The advancement of AI makes me concerned about my future employment prospects	Adapted from (Wang and Wang 2019)
	AA4	I feel uncomfortable relying heavily on AI systems in accounting work	Adapted from (Wang and Wang 2019)
Cognitive adaptability (CA)	CA1	I can easily adjust to new accounting technologies and digital systems	Haynie et al., 2010
	CA2	I modify my work strategies when technological changes occur	Adapted from (Haynie et al. 2010)
	CA3	I am willing to learn and apply AI-related accounting tools in my work	Adapted from (Haynie et al. 2010)
	CA4	I adapt quickly to changing digital accounting environments	Adapted from (Haynie et al. 2010)
Perceived AI substitution (PAIS)	PAIS1	AI can effectively perform repetitive accounting tasks traditionally undertaken by accountants	Self-developed based on (Moll and Yigitbasioglu 2019); (Sutton et al. 2016)
	PAIS2	AI can substitute analytical accounting activities that rely on data processing and pattern recognition	Self-developed based on (Moll and Yigitbasioglu 2019); (Sutton et al. 2016)
	PAIS3	AI can support and perform selected decision-support accounting functions in the future	Self-developed based on (Moll and Yigitbasioglu 2019); (Sutton et al. 2016)
	PAIS4	AI will increasingly substitute selected accounting tasks within future accounting frameworks	Self-developed based on (Moll and Yigitbasioglu 2019); (Sutton et al. 2016)

In the proposed structural model, Perceived AI Substitution (PAIS) was specified as the primary dependent variable because the study sought to explain accounting professionals' perceptions regarding AI's potential to substitute selected accounting tasks. Psychological Trust (PT) and AI Anxiety (AA) were treated as independent variables representing positive and negative psychological antecedents of AI-related perceptions. Cognitive Adaptability (CA) functioned as both a dependent variable, influenced by Psychological Trust and AI Anxiety, and as a mediating variable linking Psychological Trust to Perceived AI Substitution. This specification is consistent with the theoretical assumptions derived from the Technology Acceptance Model (TAM) and Trust Theory.

### 3.7 Reliability and validity

The reliability and validity of the measurement model were assessed using Partial Least Squares Structural Equation Modeling (PLS-SEM) through SmartPLS 4. Because the study relied on self-reported data collected through structured questionnaires, both procedural and statistical approaches were employed to establish measurement quality and minimize potential sources of bias.

Content validity was established through multiple stages. First, measurement items for Psychological Trust, AI Anxiety, and Cognitive Adaptability were adapted from previously validated scales reported in the literature. The self-developed Perceived AI Substitution (PAIS) construct was derived from the AI-accounting literature to capture accounting professionals' subjective perceptions regarding AI's capability to perform selected accounting tasks within future accounting environments. Second, the questionnaire was reviewed by three accounting academics and two industry practitioners with expertise in accounting technologies and quantitative research methods to assess conceptual clarity, relevance, and contextual appropriateness. Third, a pilot study involving 40 accounting professionals purposively recruited from the target population, representing diverse accounting-related job roles, including auditors, tax consultants, finance managers, financial analysts, and accounting academics was conducted prior to the main survey. Feedback obtained from the pilot phase resulted in minor modifications to wording and sequencing, thereby improving readability and reducing ambiguity. These procedures provide confidence that the instrument adequately captured the theoretical dimensions represented by each construct. To ensure that the instrument was comprehensible across different demographic groups, participants from various age categories and levels of professional experience were included in the pilot sample. This diversity enabled the researchers to evaluate whether the questionnaire items were interpreted consistently by respondents with differing occupational backgrounds and career stages. The pilot study confirmed that the questionnaire items adequately represented the intended constructs and were understandable to the target respondents. The refinement process enhanced the content validity and face validity of the instrument and increased confidence that the final questionnaire was suitable for large-scale data collection among accounting professionals in China.

Because all primary constructs were measured using self-reported survey responses collected from the same respondents at a single point in time, the potential influence of common method bias (CMB) was carefully considered. Following the recommendations of (Podsakoff et al. 2003), both procedural and statistical remedies were employed.

Procedurally, several design features were incorporated to reduce the likelihood of common method variance. Respondents were assured of complete anonymity and confidentiality, informed that there were no right or wrong answers, and encouraged to provide honest responses. These procedures were intended to reduce evaluation apprehension and social desirability bias. In addition, the questionnaire underwent expert review and pilot testing to improve item clarity, readability, and comprehensibility. The ordering of questionnaire sections was structured to separate demographic questions from the substantive construct measures, thereby reducing respondents' tendency to infer relationships among variables. Furthermore, construct items were presented in mixed rather than construct-by-construct blocks to reduce consistency motifs and pattern responding. The questionnaire employed simple and unambiguous wording to minimize item misinterpretation and response ambiguity.

Given that all constructs were measured using the same survey instrument, statistical assessments were also conducted. First, Harman's single-factor test was performed. The results indicated that the first unrotated factor accounted for 37.63% of the total variance, which is below the commonly recommended threshold of 50%, suggesting that no single factor dominated the covariance among the measures (Harman, 1976). However, because Harman's test is widely regarded as a relatively weak diagnostic, additional analyses were conducted. Following (Kock 2015), full collinearity variance inflation factors (VIFs) were examined for all latent constructs. The obtained VIF values ranged from 1.42 to 2.67, remaining well-below the conservative threshold of 3.30 and substantially below the maximum acceptable value of 5.00. These results indicate that common method inflation and multicollinearity were unlikely to threaten the validity of the structural estimates.

Taken together, the procedural safeguards and statistical diagnostics provide evidence that common method bias was unlikely to materially affect the findings. Nevertheless, because the study relied on cross-sectional self-reported data, the possibility of some residual method variance cannot be completely excluded. Future research may strengthen causal inference by employing longitudinal designs, multi-source data collection, or temporally separated measurements.

The reliability and validity assessment of the reflective measurement model was performed prior to evaluating the structural relationships. Internal consistency reliability was examined using Cronbach's alpha (α) and Composite Reliability (CR). As presented in Table 4, Cronbach's alpha values ranged from 0.876 to 0.902, while composite reliability values ranged from 0.907 to 0.926. All values exceeded the recommended threshold of 0.70, thereby confirming satisfactory internal consistency reliability (Hair et al., 2022). Convergent validity was assessed using indicator loadings and Average Variance Extracted (AVE). All factor loadings exceeded the recommended threshold value of 0.708, indicating that each indicator adequately represented its underlying construct. Moreover, AVE values ranged from 0.710 to 0.759, exceeding the recommended minimum criterion of 0.50 (Fornell and Larcker, 1981). These findings demonstrate that the constructs possessed satisfactory convergent validity.

**Table 4:** Reliability and validity assessment of the measurement model.

Constructs	Items	AVE	√AVE	Cronbach's alpha	Loadings	CR	VIF
Psychological trust (PT)	PT1	0.759	0.871	0.902	0.851	0.926	2.14
	PT2				0.887		2.36
	PT3				0.873		2.52
	PT4				0.865		1.94
AI anxiety (AA)	AA1	0.739	0.860	0.889	0.842	0.919	2.48
	AA2				0.857		2.31
	AA3				0.876		2.79
	AA4				0.859		2.07
Cognitive adaptability (CA)	CA1	0.710	0.843	0.876	0.824	0.907	1.85
	CA2				0.851		2.12
	CA3				0.839		2.28
	CA4				0.857		1.96
Perceived AI substitution (PAIS)	PAIS1	0.748	0.865	0.894	0.854	0.922	2.41
	PAIS2				0.872		2.67
	PAIS3				0.864		2.35
	PAIS4				0.876		2.54

AVE, Average Variance Extracted; √AVE, square root of the Average Variance Extracted; α, Cronbach's alpha; CR, composite reliability; VIF, variance inflation factor. Indicator loadings represent standardized factor loadings. Values of α and CR above 0.70 indicate satisfactory internal consistency reliability; AVE values above 0.50 indicate convergent validity; VIF values below 3.30 suggest the absence of problematic multicollinearity and severe common method bias.

Given that Perceived AI Substitution (PAIS) represented a self-developed construct, additional validation procedures were undertaken. The construct was subjected to the same reliability and validity assessments as the adapted measures. The results demonstrated satisfactory psychometric properties, with indicator loadings exceeding 0.70, AVE exceeding 0.50, and reliability coefficients surpassing recommended thresholds. These findings provide empirical support for the validity and reliability of the PAIS scale within the accounting context.

Beyond conventional reliability and convergent validity assessments (as presented in Tables 4, 5), additional analyses were conducted to evaluate the preliminary validity of the newly developed PAIS construct. The construct demonstrated satisfactory internal consistency, convergent validity, and discriminant validity according to established PLS-SEM criteria. However, these statistical indicators could be interpreted as evidence of preliminary psychometric adequacy rather than definitive construct validation. Given the exploratory nature of PAIS, the current findings represent an initial validation effort. Future research could further examine the dimensionality of the construct using exploratory and confirmatory factor analyses across independent samples, assess measurement invariance across contexts, and investigate criterion-related validity through relationships with external constructs such as AI literacy, automation readiness, occupational identity, perceived job insecurity, and technology usage behaviors.

**Table 5:** Heterotrait-monotrait (HTMT) correlation ratios.

Constructs	PT	AA	CA	PAIS
PT	–			
AA	0.462	–		
CA	0.583	0.417	–	
PAIS	0.684	0.482	0.611	–

HTMT, Heterotrait–monotrait ratio of correlations; PT, psychological trust; AA, AI anxiety; CA, cognitive adaptability; PAIS, perceived AI substitution. HTMT values below the recommended threshold of 0.85 indicate satisfactory discriminant validity among the latent constructs.

Discriminant validity was assessed using both the Fornell-Larcker criterion and the Heterotrait-Monotrait Ratio (HTMT), consistent with current best practices in PLS-SEM. The HTMT values presented in Table 5 were below the recommended threshold value of 0.85, confirming that the constructs were empirically distinct from one another (Henseler et al., 2015). The Fornell-Larcker criterion further supported discriminant validity, as the square root of each construct's AVE exceeded its correlations with other constructs (Table 4).

Data normality was also examined using skewness and kurtosis statistics (Table 6). All observed values fell within the acceptable range of ±2, indicating approximate univariate normality (Kline, 2017). Although PLS-SEM does not require strict normality assumptions, these results suggest that the data distribution did not exhibit substantial deviations from normality. Furthermore, Mahalanobis distance diagnostics indicated that no multivariate outliers were present in the dataset. Table 6 indicates that accounting professionals reported relatively high levels of psychological trust (*M* = 4.12) and cognitive adaptability (*M* = 4.08), suggesting positive orientations toward working with AI-enabled accounting systems. Perceived AI substitution also exhibited a moderately high mean (*M* = 3.87), reflecting a belief that AI may increasingly perform selected accounting tasks in future accounting frameworks. In contrast, AI anxiety demonstrated comparatively lower mean values (*M* = 3.41), indicating the presence of concerns regarding automation and occupational change without overwhelming resistance. Furthermore, all skewness and kurtosis values remained within acceptable thresholds (±2), supporting the assumption of approximate normality and confirming the suitability of the data for subsequent PLS-SEM analysis.

**Table 6:** Descriptive statistics.

Construct	Mean	SD	Skewness	Kurtosis
Psychological trust (PT)	4.12	0.67	−0.38	0.14
AI anxiety (AA)	3.41	0.82	−0.19	−0.24
Cognitive adaptability (CA)	4.08	0.63	−0.31	0.09
Perceived AI substitution (PAIS)	3.87	0.71	−0.27	−0.11

SD, standard deviation. Skewness and kurtosis values within ±2 indicate approximate univariate normality of the observed data distribution.

Potential endogeneity concerns associated with the cross-sectional and perception-based nature of the study were also considered. Endogeneity may arise due to omitted variables, measurement error, or reciprocal relationships among latent constructs. To mitigate these concerns, the proposed model was developed based on a clear theoretical rationale grounded in Technology Acceptance Model (TAM), Trust Theory, and contemporary psychological perspectives on occupational adaptation. Multicollinearity diagnostics confirmed that predictor constructs did not exhibit problematic overlap. Alternative model specifications involving reversed causal directions were also examined, and the hypothesized model demonstrated stronger explanatory capability than theoretically plausible alternatives. Although the cross-sectional design precludes definitive causal inference, these diagnostic procedures reduce the likelihood that the observed relationships are artifacts of model misspecification.

To assess the overall adequacy of the proposed model, model fit was evaluated using the Standardized Root Mean Square Residual (SRMR) and the Normed Fit Index (NFI). The SRMR value was 0.067, which falls below the recommended threshold of 0.08, indicating a satisfactory fit between the observed data and the estimated model (Hu and Bentler, 1999). In addition, the Normed Fit Index (NFI) was 0.912, exceeding the commonly accepted minimum criterion of 0.90 for acceptable model fit (Hair et al., 2022). Collectively, these indices suggest that the measurement and structural models exhibit adequate fit and that the proposed PLS-SEM model provides a satisfactory representation of the underlying data.

### 3.8 Data analysis technique

The quantitative data were analyzed using Partial Least Squares Structural Equation Modeling (PLS-SEM) through SmartPLS software (SmartPLS GmbH, Germany). PLS-SEM was selected because it is suitable for predictive research models involving complex relationships, mediation analysis, and latent psychological constructs. Additionally, PLS-SEM does not require strict normality assumptions and is highly appropriate for exploratory and theory-development studies. The quantitative analysis followed a two-stage analytical procedure consisting of measurement model assessment and structural model assessment.

The qualitative phase of the study sought to explain and contextualize the statistical relationships identified through the PLS-SEM analysis. Following the explanatory sequential mixed-method design, semi-structured interviews were conducted with 25 accounting professionals, including auditors, finance managers, tax consultants, accounting academics, and accounting technology specialists. The interviews explored participants' experiences and perceptions regarding the increasing integration of AI within accounting functions and their views concerning AI's potential to substitute selected accounting tasks. The qualitative data were analyzed using thematic analysis following the procedures recommended by (Clarke 2006). The analysis involved four stages. First, the interview transcripts were repeatedly reviewed to achieve familiarity with the data. Second, meaningful statements and recurring ideas were identified through open coding. Third, conceptually similar codes were grouped into broader second-order themes. Finally, these themes were integrated into aggregate dimensions corresponding to the major constructs examined quantitatively: psychological trust, AI anxiety, cognitive adaptability, and perceived AI substitution. This systematic coding procedure enhanced analytical transparency and strengthened the credibility and trustworthiness of the qualitative findings.

The coding structure presented in Table 7 demonstrates how participants' narratives progressed from raw textual evidence to higher-order conceptual interpretations. The aggregate dimensions closely aligned with the PLS-SEM findings and facilitated the development of meta-inferences regarding the psychological processes underlying perceptions of AI substitution in accounting contexts. The coding indicates that trust in AI emerged through perceptions of competence and transparency, whereas AI anxiety reflected concerns regarding job insecurity and professional identity. Cognitive adaptability highlighted the importance of continuous learning and flexibility, while perceived AI substitution emphasized selective task substitution and human–AI complementarity. This transparent coding process strengthens the credibility and trustworthiness of the mixed-method findings.

**Table 7:** Qualitative coding structure.

Representative quotations	First-order codes	Second-order themes	Aggregate dimensions
“AI can process transactions and reconcile accounts much faster than humans”	Efficiency; accuracy; reliability	Confidence in AI capabilities	Psychological trust
“I trust AI for routine tasks, but transparency remains essential”	Reduced errors; explainability	Trust through accountability	Psychological trust
“Younger accountants may lose opportunities because automation is expanding rapidly”	Job insecurity; fear of replacement	Perceived professional threat	AI anxiety
“People worry whether their skills will remain relevant”	Skill obsolescence; Career uncertainty	Threat to professional identity	AI anxiety
“We need to continuously learn and adapt our work practices”	Continuous learning; flexibility	Adaptive learning orientation	Cognitive adaptability
“AI requires accountants to develop new competencies”	Upskilling; role adjustment	Professional adaptation	Cognitive adaptability
“Ethical judgments still require human involvement”	Ethical reasoning; communication	Human–AI complementarity	Perceived AI substitution
“AI supports rather than completely replaces accountants”	Strategic judgment; contextual understanding	Selective substitution	Perceived AI substitution

To enhance the transparency and credibility of the qualitative findings, representative quotations from the semi-structured interviews are presented in Table 8. The quotations provide direct evidence of participants' experiences and perceptions regarding the increasing integration of artificial intelligence (AI) within accounting practices. Presenting verbatim excerpts allows readers to assess the extent to which the qualitative interpretations accurately reflect participants' perspectives and supports the trustworthiness of the thematic analysis. The interview excerpts reveal that accounting professionals hold nuanced and sometimes competing views regarding AI-enabled transformation. While many participants acknowledged the efficiency and analytical benefits of AI technologies, they simultaneously expressed concerns related to professional identity, employability, and the continuing importance of human judgment. These findings complement the PLS-SEM results by explaining the psychological mechanisms underlying the observed statistical relationships.

**Table 8:** Illustrative interview quotations.

Theme	Illustrative interview quotations
Psychological trust	“AI can process large volumes of financial information with remarkable speed and accuracy. If the outputs remain transparent and verifiable, I am comfortable relying on these systems for routine accounting activities.” (Finance Manager, 14 years of experience)
Psychological trust	“I trust AI when it enhances the quality of our work rather than replacing professional judgment. Reliability and accountability are essential for acceptance.” (Auditor, 11 years of experience)
AI anxiety	“Many accountants worry that younger professionals may struggle to establish their careers because automation is rapidly changing the profession.” (Tax Consultant, 16 years of experience)
AI anxiety	“The fear is not the technology itself; it is uncertainty about whether our expertise will still be valued in the future.” (Accounting Academic, 19 years of experience)
Cognitive adaptability	“We cannot avoid technological change. Continuous learning and updating our skills have become part of being an accountant.” (Senior Accountant, 12 years of experience)
Cognitive adaptability	“AI requires us to think differently. Instead of focusing on repetitive tasks, we need to develop analytical and interpretative capabilities.” (Financial Analyst, 9 years of experience)
Perceived AI substitution	“AI is highly effective in handling routine processes such as reconciliation and transaction verification, but ethical decisions still require human judgment.” (Auditor, 17 years of experience)
Perceived AI substitution	“I do not believe AI will completely replace accountants. Rather, it will redefine our roles and allow us to focus on strategic responsibilities.” (Finance Executive, 21 years of experience)

Furthermore, inter-coder reliability was assessed during the coding process. Two trained researchers independently conducted open coding, axial coding, and selective coding of the interview transcripts following the established coding framework. Their role was limited to reviewing coding interpretations and providing feedback on theme boundaries rather than acting as co-authors or making analytical decisions. The coding outcomes were subsequently compared, and Cohen's Kappa coefficient was calculated to evaluate the degree of agreement between the coders. The obtained Cohen's Kappa value exceeded 0.80, indicating a high level of coding consistency and substantial agreement according to widely accepted benchmarks (Landis and Koch, 1977). Any discrepancies identified during the coding process were resolved through discussion and consensus, thereby strengthening the reliability and credibility of the qualitative findings.

The study strictly adhered to ethical research principles throughout the research process. Participants were informed regarding the purpose of the study, confidentiality of responses, anonymity protection, and voluntary participation rights. Informed consent was obtained from all respondents before participation. Participants were assured that collected information would be used solely for academic purposes and would not be disclosed to third parties. Additionally, respondents were allowed to withdraw from the study at any stage without penalty. Interview recordings and survey responses were securely stored and accessed only by the researchers to maintain data privacy and confidentiality.

## 4 Results and analysis

### 4.1 Structural model assessment

The structural model was assessed using Partial Least Squares Structural Equation Modeling (PLS-SEM) through SmartPLS 4. The analysis examined the direct relationships among psychological trust, AI anxiety, cognitive adaptability, and perceptions regarding artificial intelligence (AI) substitution within future accounting frameworks. The model also evaluated the mediating role of cognitive adaptability between psychological trust and AI substitution perception. Bootstrapping with 5,000 subsamples was performed to determine the significance of the hypothesized relationships.

The structural model demonstrated satisfactory explanatory power for the endogenous constructs. The coefficient of determination (*R*^2^) value for Cognitive Adaptability was 0.514, indicating that Psychological Trust and AI Anxiety jointly explained 51.4% of the variance in accounting professionals' cognitive adaptability. According to (Hair et al. 2022), *R*^2^ values exceeding 0.50 reflect moderate to substantial explanatory capability, suggesting that the proposed antecedents provide a meaningful explanation of professionals' adaptive responses toward AI-enabled accounting environments. More importantly, the *R*^2^ value for Perceived AI Substitution was 0.674, indicating that Psychological Trust, AI Anxiety, and Cognitive Adaptability collectively accounted for 67.4% of the variance in perceptions regarding AI's potential to substitute selected accounting tasks. This relatively high explanatory power suggests that the proposed model effectively captures the major psychological determinants underlying accounting professionals' evaluations of AI-enabled transformation within the Chinese accounting context.

The predictive relevance of the structural model was further assessed using Stone–Geisser's *Q*^2^ values obtained through the blindfolding procedure. The *Q*^2^ values for Cognitive Adaptability (*Q*^2^ = 0.381) and Perceived AI Substitution (*Q*^2^ = 0.472) were both greater than zero. Following the recommendations of (Hair et al. 2022), *Q*^2^ values above zero indicate that the model possesses predictive relevance and is capable of accurately reproducing observed data patterns for omitted cases. Therefore, the findings verify the satisfactory out-of-sample predictive ability of the structural model, demonstrating that the proposed framework not only explains existing relationships among the constructs but also exhibits adequate capability in predicting accounting professionals' perceptions and adaptive responses toward AI integration.

The structural path analysis was conducted using Partial Least Squares Structural Equation Modeling (PLS-SEM) to examine the direct relationships among psychological trust, AI anxiety, cognitive adaptability, and AI substitution perception within the future accounting framework (Table 9). The significance of the hypothesized relationships was evaluated using path coefficients (β), *t*-values, and *p*-values obtained through bootstrapping with 5,000 resamples. The results provide strong empirical support for the proposed conceptual framework and demonstrate the critical role of psychological factors in shaping perceptions regarding artificial intelligence (AI) substitution in accounting professions.

**Table 9:** Structural path results.

Hypotheses	Structural path	Beta (β)	Standard deviation	*t*-Value	*p*-Value	Decision
H1	Psychological trust → AI substitution perception	0.432	0.048	8.921	0.000	Supported
H2	AI anxiety → AI substitution perception	−0.318	0.047	6.774	0.000	Supported
H3	Cognitive adaptability → AI substitution perception	0.389	0.052	7.456	0.000	Supported
H4	Psychological trust → Cognitive adaptability	0.471	0.051	9.116	0.000	Supported
H5	AI anxiety → Cognitive adaptability	−0.294	0.050	5.882	0.000	Supported

β, standardized path coefficient; SD, standard deviation; *t*-value, bootstrap *t* statistic; *p*-value, significance probability derived from bootstrapping; Bootstrap subsamples, 5,000. R^2^ values for Cognitive Adaptability and Perceived AI Substitution were 0.514 and 0.674, respectively. Q^2^ values were 0.381 and 0.472, respectively, indicating satisfactory predictive relevance. p < 0.001.

Source: Primary.

The analysis revealed that psychological trust exerted a significant positive influence on AI substitution perception (β = 0.432, *t* = 8.921, *p* < 0.001), thereby supporting Hypothesis 1. The positive beta coefficient indicates that higher levels of trust in AI systems substantially increase accounting professionals' perceptions that AI can effectively substitute human accounting tasks. The large *t*-value and highly significant *p*-value confirm the robustness and statistical significance of this relationship. This finding suggests that trust functions as one of the most influential psychological determinants encouraging acceptance of AI-enabled accounting systems. Accounting professionals who perceive AI technologies as reliable, transparent, competent, and accurate are more willing to rely on intelligent systems for performing accounting functions such as auditing, financial reporting, fraud detection, and predictive analytics. The result aligns with Trust Theory, which argues that trust reduces uncertainty and enhances users' willingness to depend on autonomous technological systems (Mayer et al., 1995). The finding further indicates that trust is essential for facilitating digital transformation within accounting environments because professionals are unlikely to accept AI substitution unless they possess confidence in the reliability and integrity of intelligent systems.

Furthermore, the result is consistent with the Technology Acceptance Model (TAM), whereby trust strengthens perceptions of usefulness and facilitates acceptance of technological innovations. Within the Chinese accounting context, this finding assumes particular significance. China's accounting profession operates within a highly regulated environment characterized by strong compliance requirements and standardized procedures. Many accounting activities, such as invoice processing, reconciliation, compliance monitoring, and routine auditing procedures, follow structured protocols. Under such conditions, trust in AI-generated outputs reduces uncertainty and encourages professionals to view AI systems as legitimate tools capable of assuming routine accounting responsibilities. Therefore, trust emerges as the core psychological foundation supporting acceptance of AI-enabled transformation in accounting practice.

The results also demonstrated that AI anxiety had a significant negative influence on AI substitution perception (β = −0.318, *t* = 6.774, *p* < 0.001), supporting Hypothesis 2. The negative beta coefficient indicates that increased feelings of fear, uncertainty, discomfort, and insecurity regarding AI technologies reduce professionals' acceptance of AI substitution in accounting functions. This finding highlights that psychological resistance toward AI remains a major challenge within technologically transforming accounting environments. Accounting professionals who fear job displacement, technological dependency, skill obsolescence, and loss of professional relevance are less likely to support the integration of AI-enabled systems. The significant *t*-value confirms that AI anxiety substantially weakens positive attitudes toward intelligent accounting technologies. This result supports prior studies emphasizing that technological anxiety negatively affects technology acceptance and innovation adoption behavior (Wang and Wang, 2019). Within the Chinese accounting context, the rapid automation of routine accounting tasks may intensify professionals' fears regarding employment insecurity, thereby generating resistance toward AI substitution. The finding suggests that organizations implementing intelligent accounting systems must address employees' psychological concerns through training, communication, and technological support initiatives to reduce anxiety and facilitate smoother AI adoption. Consistent with organizational psychology literature, technology-related fear undermines positive evaluations of innovation and increases resistance to change. Professionals who perceive AI as threatening their occupational security tend to reject narratives emphasizing technological substitution, even when recognizing AI's operational capabilities. The result therefore highlights the importance of addressing technology-related fears through communication, reskilling initiatives, and organizational support mechanisms.

Cognitive adaptability was found to positively and significantly influence AI substitution perception (β = 0.389, *t* = 7.456, *p* < 0.001), thereby supporting Hypothesis 3. The result indicates that accounting professionals possessing stronger adaptability toward technological change are more likely to perceive AI as capable of substituting repetitive and analytical accounting tasks. Cognitive adaptability reflects individuals' ability to modify cognitive strategies, learning behaviors, and problem-solving approaches in response to changing technological environments. The positive beta coefficient suggests that adaptable professionals demonstrate greater openness toward AI integration because they can more effectively learn intelligent systems, adjust to digital accounting processes, and integrate AI tools into professional activities. The finding highlights the importance of adaptive capabilities in technologically evolving workplaces. Accounting professionals who possess flexible learning orientations are less threatened by technological transformation and more willing to collaborate with intelligent systems. This result is consistent with previous research indicating that adaptability enhances technological readiness and innovation acceptance behavior (Haynie et al., 2010). The finding further suggests that cognitive adaptability is critical for sustaining workforce relevance in future AI-driven accounting environments. This finding reinforces the Technology Acceptance Model by suggesting that adaptability enhances individuals' ability to perceive AI systems as manageable and beneficial. Adaptable professionals are more capable of integrating AI-generated insights into their work routines and redefining their professional roles within technologically evolving environments.

The analysis further revealed that psychological trust significantly and positively influenced cognitive adaptability (β = 0.471, *t* = 9.116, *p* < 0.001), thereby supporting Hypothesis 4. This relationship demonstrated the strongest positive path coefficient among all structural relationships in the model. The finding indicates that accounting professionals who trust AI systems are more likely to develop adaptive cognitive behaviors toward technological transformation. Trust encourages individuals to engage positively with intelligent systems, participate in technological learning processes, and experiment with AI-enabled accounting tools. When professionals perceive AI systems as reliable and beneficial, they become more willing to modify existing accounting practices and adapt to technologically advanced work environments. The high *t*-value confirms the substantial importance of trust in shaping adaptive technological behavior. This finding reinforces the argument that trust not only directly influences AI substitution perceptions but also indirectly strengthens professionals' ability to adapt cognitively to AI-driven accounting transformation. The finding suggests that trust functions not only as a direct predictor of AI acceptance but also as an enabling psychological condition that promotes adaptive learning behaviors. Consistent with Trust Theory, confidence in technological systems reduces uncertainty and facilitates engagement with new work practices. In the context of TAM, trusted technologies are perceived as more useful and less intimidating, thereby motivating professionals to invest cognitive effort in mastering AI-enabled systems.

The results additionally showed that AI anxiety negatively influenced cognitive adaptability (β = −0.294, *t* = 5.882, *p* < 0.001), thereby supporting Hypothesis 5. The negative beta coefficient indicates that professionals experiencing higher levels of fear and uncertainty regarding AI technologies demonstrate lower adaptability toward intelligent accounting systems. Anxiety creates psychological barriers that discourage technological learning, reduce confidence in engaging with intelligent systems, and limit professionals' willingness to modify cognitive strategies. The finding suggests that fear of automation negatively affects professionals' capacity to adapt to digital transformation within accounting environments. Professionals who perceive AI as threatening may avoid learning AI-related competencies and resist organizational technological initiatives. The significant relationship highlights the detrimental impact of technological anxiety on adaptive behavior and workforce transformation readiness. This result is particularly relevant in the Chinese accounting sector, where rapid AI implementation may create significant psychological pressure among professionals unfamiliar with intelligent technologies. Organizational psychology literature suggests that fear constrains learning orientation and reduces behavioral flexibility. Therefore, reducing AI-related anxiety represents an important prerequisite for enhancing adaptive capacity among accounting professionals.

Overall, the structural path results demonstrate that psychological trust and cognitive adaptability are significant positive drivers of AI substitution perception, whereas AI anxiety functions as a major psychological barrier. Among all predictors, psychological trust exhibited the strongest influence on both AI substitution perception and cognitive adaptability, emphasizing the importance of reliability, confidence, and transparency in facilitating AI adoption within accounting systems. The findings collectively suggest that successful AI integration in future accounting frameworks depends not only on technological capabilities but also on professionals' psychological readiness, emotional responses, and adaptive competencies. The results further indicate that AI can effectively substitute repetitive, rule-based, and analytical accounting tasks; however, professionals' acceptance of AI-enabled accounting systems largely depends on whether organizations can build trust, reduce technological anxiety, and enhance cognitive adaptability among employees. Consequently, accounting firms, policymakers, and AI developers must prioritize psychological support mechanisms, continuous technological training, and adaptive learning initiatives to ensure sustainable and human-centered AI transformation within accounting professions.

#### 4.1.1 Mediation analysis

The mediation analysis was conducted using the bootstrapping procedure in SmartPLS with 5,000 resamples to examine whether Cognitive Adaptability mediates the relationship between Psychological Trust and Perceived AI Substitution. The results presented in Table 10 indicate that the indirect effect of Psychological Trust on Perceived AI Substitution through Cognitive Adaptability was positive and statistically significant (β = 0.184, *t* = 4.911, *p* < 0.001). Therefore, Hypothesis H6 was supported.

**Table 10:** Mediation analysis.

Hypothesis	Indirect path	β	SD	*t*-Value	*p*-Value	VAF	Decision
H6	Psychological trust → Cognitive adaptability → AI substitution perception	0.184	0.037	4.911	0.000	29.87%	Supported

β, standardized indirect effect; SD, standard deviation; *t*-value, bootstrap *t* statistic; *p*-value, significance probability derived from bootstrapping. Indirect effects were assessed using 5,000 bootstrap subsamples. A significant indirect effect indicates mediation within the proposed structural model. The Variance Accounted For (VAF) was 29.87%, calculated as the ratio of the indirect effect (β = 0.184) to the total effect (β = 0.616). p < 0.001.

Source: Primary.

To further assess the strength of the mediating effect, the Variance Accounted For (VAF) was calculated by dividing the indirect effect by the total effect. The total effect of Psychological Trust on Perceived AI Substitution was 0.616, comprising a direct effect of 0.432 and an indirect effect of 0.184. The resulting VAF value was 29.87% [(0.184/0.616) × 100], indicating that approximately 30% of the total influence of Psychological Trust on Perceived AI Substitution operated through Cognitive Adaptability. Following the guidelines proposed by (Hair et al. 2022), VAF values between 20 and 80% indicate partial mediation. Thus, Cognitive Adaptability partially mediates the relationship between Psychological Trust and Perceived AI Substitution.

The mediation findings also provide deeper insights into the underlying psychological mechanism through which accounting professionals form perceptions regarding AI-enabled transformation. Specifically, the results reveal a sequential psychological pathway whereby changes in individual psychological attitudes precede cognitive adjustment, which subsequently shapes perceptions of AI substitution. Accounting professionals who develop greater trust in AI systems initially experience more favorable psychological evaluations of intelligent technologies. These positive attitudes reduce uncertainty and encourage openness toward technological engagement. Such psychological confidence subsequently promotes cognitive adjustment, enabling professionals to modify their thinking patterns, acquire new technological competencies, and adapt their work strategies to AI-enabled environments. Through this adaptive process, professionals become more willing to perceive AI as capable of substituting selected routine and analytical accounting tasks.

This chain mechanism extends the explanatory power of both the Technology Acceptance Model (TAM) and Trust Theory by demonstrating that trust influences perceptions of AI substitution not only directly but also indirectly through adaptive cognitive processes (Davis, 1989; Mayer et al., 1995). Rather than representing a simple technological acceptance response, perceptions of AI substitution emerge through a psychologically embedded process involving attitudinal evaluation, cognitive adaptation, and the subsequent reinterpretation of professional roles. This interpretation is consistent with contemporary perspectives on occupational adaptation, which suggest that professionals actively reconstruct their identities and competencies in response to technological disruption (Savickas, 2013), as well as emerging research on human–AI collaboration emphasizing that intelligent systems reshape rather than merely replace professional work (Dellermann et al., 2019; Dwivedi et al., 2023). The findings therefore contribute theoretically by identifying Cognitive Adaptability as a key psychological transmission mechanism linking trust in intelligent systems to acceptance of AI-enabled occupational transformation, thereby extending traditional technology acceptance models beyond direct attitudinal effects to include adaptive psychological processes (Haynie et al., 2010; Jöhnk et al., 2021). From a practical perspective, the results suggest that organizations seeking to facilitate AI integration should not only strengthen employees' trust in intelligent systems but also invest in adaptive learning initiatives, continuous reskilling programmes, and technological capability development to enhance workforce readiness for AI-driven change, consistent with recent recommendations on developing AI literacy and adaptive competencies in professional settings (Ng et al., 2021; Kelly et al., 2023).

#### 4.1.2 Effect size analysis

The effect size (*f*^2^) analysis was conducted to assess the practical contribution of each exogenous construct to the endogenous variables in the structural model (Table 11). Following (Cohen 1988), *f*^2^ values of 0.02, 0.15, and 0.35 indicate small, medium, and large effect sizes, respectively. While statistical significance demonstrates whether relationships exist, effect sizes provide insight into the relative importance and practical significance of each predictor.

**Table 11:** Effect size (*f* ^2^) analysis.

Structural relationship	*f*^2^ value	Effect size
Psychological trust → AI substitution perception	0.267	Medium
AI anxiety → AI substitution perception	0.192	Medium
Cognitive adaptability → AI substitution perception	0.241	Medium

f^2^ represents the effect size of each exogenous construct on the endogenous construct. Following (Cohen 1988), f^2^ values of 0.02, 0.15, and 0.35 indicate small, medium, and large effect sizes, respectively.

Source: Primary.

The findings revealed that Psychological Trust exerted a medium effect on Perceived AI Substitution (*f*^2^ = 0.267), indicating that trust represents one of the most influential drivers shaping accounting professionals' beliefs regarding AI's capability to perform selected accounting tasks. Professionals who perceive AI systems as reliable, transparent, and competent are substantially more likely to support the integration of AI into accounting functions. This finding suggests that organizations seeking to increase acceptance of AI-enabled accounting systems should prioritize initiatives that strengthen trust through system transparency, explainability, accountability, and demonstrated reliability.

Cognitive Adaptability also demonstrated a medium effect on Perceived AI Substitution (*f*^2^ = 0.241). This result indicates that professionals' ability to adjust their thinking patterns, learn new technological competencies, and adapt to evolving work practices meaningfully contributes to positive perceptions of AI substitution. The finding highlights that acceptance of AI is not solely dependent on technological characteristics but also on employees' readiness to adapt to digital transformation. Consequently, continuous learning opportunities, reskilling programmes, and adaptive training interventions should be emphasized within accounting organizations.

Similarly, AI Anxiety exhibited a medium negative effect on Perceived AI Substitution (*f*^2^ = 0.192), suggesting that concerns regarding job insecurity, professional obsolescence, and technological uncertainty constitute important psychological barriers to AI acceptance. Although the effect was weaker than the positive influence of trust, it remains practically meaningful. This finding implies that organizations implementing intelligent accounting systems should actively address employees' fears through transparent communication, career development support, and change-management initiatives designed to reduce resistance toward technological transformation.

More importantly, Psychological Trust produced a large effect on Cognitive Adaptability (*f*^2^ = 0.314), representing the strongest practical influence observed in the structural model. This finding suggests that trust functions as the core factor enhancing practitioners' adaptability toward AI-enabled environments. Accounting professionals who trust intelligent systems are more willing to experiment with new technologies, modify established work routines, and acquire AI-related competencies. Therefore, fostering trust in AI systems extends beyond promoting acceptance; it fundamentally strengthens the workforce's capacity to adapt to technological change.

In contrast, AI Anxiety exerted a comparatively weaker negative effect on Cognitive Adaptability (*f*^2^ = 0.143). Although this effect approached the threshold for a medium effect, it suggests that anxiety constrains adaptive behavior to a lesser extent than trust facilitates it. Taken together, the effect size analysis demonstrates that positive psychological resources, particularly trust, play a more influential role in shaping technological adaptation than negative emotional responses. These findings carry important practical implications, indicating that accounting firms, policymakers, and AI developers should prioritize trust-building mechanisms and adaptive capability development rather than focusing exclusively on reducing technological fears. Such strategies are likely to promote more sustainable and human-centered AI transformation within the accounting profession.

### 4.2 Inter-group differences in core psychological variables

To provide a more comprehensive understanding of how accounting professionals respond to AI-enabled transformation, supplementary inter-group analyses were conducted using independent-samples *t*-tests and one-way ANOVA. While the PLS-SEM results explain the structural relationships among psychological trust, AI anxiety, cognitive adaptability, and perceived AI substitution, they do not indicate whether these psychological characteristics differ systematically across demographic and professional categories. Examining such differences is important because perceptions of technological disruption and adaptation are often influenced by individuals' career stage, professional experience, and occupational responsibilities. Respondents were classified according to gender, age, years of professional experience, and job positions. Independent-samples *t*-tests were employed to examine gender differences, whereas one-way ANOVA was used to compare differences among age groups, experience categories, and occupational positions. The analyses focused on the three core psychological constructs examined in this study: psychological trust, AI anxiety, and cognitive adaptability.

The results reported in Table 12 demonstrate statistically significant variations in psychological trust, AI anxiety, and cognitive adaptability across several demographic and professional groups. These findings suggest that accounting professionals are not a homogeneous population in their responses to AI-enabled transformation and that organizational strategies for AI implementation should recognize these differences.

**Table 12:** Inter-group differences in core psychological variables.

Grouping variable	*n*	Psychological trust mean (SD)	AI anxiety mean (SD)	Cognitive adaptability mean (SD)	Test statistic	*p*-Value	Effect size
Gender							
Male	276	3.87 (0.61)	3.22 (0.68)	3.94 (0.58)	*t*_(510)_ = 1.08; 4.12; 0.95	0.281; < 0.001; 0.343	*d* = 0.09; 0.37; 0.08
Female	236	3.82 (0.63)	3.47 (0.70)	3.89 (0.60)			
Age groups							
25–30 years	84	3.65 (0.66)	3.56 (0.71)	3.70 (0.63)	PT: *F*_(3, 508)_ = 9.14	< 0.001	η^2^ = 0.051
31–35 years	118	3.89 (0.60)	3.24 (0.66)	3.91 (0.58)	AA: *F*_(3, 508)_ = 13.22	< 0.001	η^2^ = 0.072
36–45 years	223	4.03 (0.56)	2.98 (0.61)	4.10 (0.54)	CA: *F*_(3, 508)_ = 10.47	< 0.001	η^2^ = 0.058
Above 45 years	87	3.78 (0.63)	3.29 (0.67)	3.74 (0.61)			
Professional experience							
Less than 5 years	98	3.62 (0.68)	3.59 (0.72)	3.67 (0.64)	PT: *F*_(4, 507)_ = 10.86	< 0.001	η^2^ = 0.079
6–10 years	154	3.79 (0.61)	3.33 (0.68)	3.88 (0.59)	AA: *F*_(4, 507)_ = 14.95	< 0.001	η^2^ = 0.106
11–15 years	128	3.95 (0.58)	3.12 (0.65)	4.01 (0.56)	CA: *F*_(4, 507)_ = 11.72	< 0.001	η^2^ = 0.085
16–20 years	77	4.06 (0.55)	2.98 (0.61)	4.11 (0.53)			
More than 20 years	55	4.14 (0.54)	2.86 (0.59)	4.18 (0.52)			
Professional role							
Financial analyst	127	3.98 (0.58)	3.05 (0.63)	4.04 (0.55)	PT: *F*_(4, 507)_ = 7.84	< 0.001	η^2^ = 0.058
Auditor	120	3.83 (0.61)	3.23 (0.65)	3.99 (0.57)	AA: *F*_(4, 507)_ = 8.67	< 0.001	η^2^ = 0.064
Finance manager	110	4.12 (0.55)	2.89 (0.59)	4.06 (0.54)	CA: *F*_(4, 507)_ = 6.92	< 0.001	η^2^ = 0.052
Tax consultant	86	3.57 (0.67)	3.58 (0.69)	3.69 (0.62)			
Accounting academics	69	3.88 (0.60)	3.16 (0.64)	4.21 (0.50)			

All Values represent means (M) and standard deviations (SD). Independent-samples *t*-tests were used to assess gender differences, whereas one-way analysis of variance (ANOVA) was employed to examine differences across age groups, professional experience levels, and professional roles.

PT, Psychological Trust; AA, AI anxiety; CA, cognitive adaptability; *t*, t-statistic; F, ANOVA F-statistic; df, degrees of freedom; *p*, probability value; *d*, Cohen's effect size for t-tests; η^2^, eta-squared effect size for ANOVA. According to (Cohen 1988), *d* values of 0.20, 0.50, and 0.80 indicate small, medium, and large effects, respectively, while η^2^ values of 0.01, 0.06, and 0.14 indicate small, medium, and large effects, respectively. Statistical significance was evaluated at *p* < 0.05. Significant ANOVA results were followed by Tukey's HSD *post-hoc* tests. Higher scores on PT and CA indicate more favorable perceptions of AI-enabled transformation, whereas higher scores on AA indicate greater concern regarding AI-related occupational disruption.

#### 4.2.1 Gender differences

The gender-based analysis revealed that male and female respondents exhibited remarkably similar levels of psychological trust and cognitive adaptability. Male respondents reported slightly higher psychological trust (*M* = 3.87, SD = 0.61) than females (*M* = 3.82, SD = 0.63), but this difference was not statistically significant, *t*_(510)_ = 1.08, *p* = 0.281, *d* = 0.09. Likewise, cognitive adaptability was nearly identical between males (*M* = 3.94, SD = 0.58) and females (*M* = 3.89, SD = 0.60), *t*_(510)_ = 0.95, *p* = 0.343, *d* = 0.08. The negligible effect sizes suggest that gender contributes very little to explaining differences in trust and adaptability toward AI technologies.

However, a statistically significant difference emerged for AI anxiety. Female respondents reported significantly higher anxiety levels (*M* = 3.47, SD = 0.70) than male respondents (*M* = 3.22, SD = 0.68), *t*_(510)_ = 4.12, *p* < 0.001, *d* = 0.37. Although the effect size falls within the small-to-moderate range, the mean difference of 0.25 points on a five-point scale indicates a meaningful divergence in emotional responses toward AI adoption. This finding suggests that while both genders recognize the usefulness of AI and demonstrate comparable adaptive capabilities, female professionals may perceive greater uncertainty regarding the consequences of technological change. Such concerns may relate to perceptions of job restructuring, increased performance expectations, or the evolving nature of professional responsibilities in AI-enabled environments. Consequently, organizations implementing AI technologies should complement technical training initiatives with communication and support mechanisms that address technology-related anxieties and reinforce employees' confidence in adapting to digital transformation.

#### 4.2.2 Age-based differences

Age emerged as a significant determinant of all three psychological constructs. The ANOVA results revealed significant differences in psychological trust, *F*_(3, 508)_ = 9.14, *p* < 0.001, η^2^ = 0.051. Respondents aged 36–45 years reported the highest trust levels (*M* = 4.03, SD = 0.56), exceeding those aged 25–30 years (*M* = 3.65, SD = 0.66) by approximately 10%. This pattern suggests that mid-career professionals may possess sufficient experience to appreciate the strategic benefits of AI while remaining receptive to technological innovation.

The strongest age-related variation was observed for AI anxiety, *F*_(3, 508)_ = 13.22, *p* < 0.001, η^2^ =0.072. Younger respondents aged 25–30 years reported the highest anxiety levels (*M* = 3.56, SD = 0.71), whereas respondents aged 36–45 years exhibited the lowest anxiety (*M* = 2.98, SD = 0.61). The difference of 0.58 points represents a substantial practical gap and indicates that younger professionals are considerably more concerned about AI-related disruption. Since early-career professionals have yet to establish strong professional identities and career security, they may perceive automation as a direct threat to future advancement opportunities.

Cognitive adaptability also varied significantly across age groups, *F*_(3, 508)_ = 10.47, *p* < 0.001, η^2^ = 0.058. Again, respondents aged 36–45 years reported the highest adaptability (*M* = 4.10, SD = 0.54), while those above 45 years demonstrated comparatively lower adaptability (*M* = 3.74, SD = 0.61). These findings suggest that middle-aged professionals occupy an advantageous position characterized by both substantial expertise and continued openness to learning. In contrast, older professionals may possess extensive technical knowledge but may face greater challenges adapting established work practices to emerging technological systems.

#### 4.2.3 Professional experience differences

Among all demographic variables examined, professional experience exhibited the strongest explanatory power. Significant differences were observed for psychological trust, *F*_(4, 507)_ = 10.86, *p* < 0.001, η^2^ = 0.079; AI anxiety, *F*_(4, 507)_ = 14.95, *p* < 0.001, η^2^ = 0.106; and cognitive adaptability, *F*_(4, 507)_ = 11.72, *p* < 0.001, η^2^ = 0.085. Notably, the effect size for AI anxiety (η^2^ = 0.106) was the largest reported in Table 12, indicating that professional experience is a particularly important determinant of emotional reactions toward AI-enabled transformation.

A clear linear pattern emerged across experience levels. Psychological trust increased steadily from 3.62 among professionals with less than 5 years of experience to 4.14 among those with more than 20 years of experience. Similarly, cognitive adaptability increased from 3.67 to 4.18, while AI anxiety declined from 3.59 to 2.86. The magnitude of these differences suggests that experienced professionals increasingly view AI as a complementary technology rather than a substitute for human expertise.

This pattern may be explained by accumulated professional confidence and specialized knowledge. Experienced professionals possess deeper contextual understanding, enabling them to recognize the limitations of AI and appreciate the continuing importance of human judgment. Consequently, they are less likely to perceive AI as a threat to their professional identity. By contrast, less experienced employees may lack comparable confidence and therefore exhibit greater concern regarding automation-related job displacement and career uncertainty.

#### 4.2.4 Professional role differences

The findings also revealed significant differences across professional roles. Psychological trust differed significantly among occupational groups, *F*_(4, 507)_ = 7.84, *p* < 0.001, η^2^ = 0.058. Finance managers reported the highest trust levels (*M* = 4.12, SD = 0.55), whereas tax consultants reported the lowest (*M* = 3.57, SD = 0.67). The difference of 0.55 points indicates a substantial divergence in how these professional groups perceive AI technologies.

Similarly, AI anxiety varied significantly across occupational categories, *F*_(4, 507)_ = 8.67, *p* < 0.001, η^2^ = 0.064. Tax consultants reported the highest anxiety (*M* = 3.58, SD = 0.69), whereas finance managers reported the lowest (*M* = 2.89, SD = 0.59). The gap of 0.69 points represents the largest occupational difference observed in the study. This result suggests that tax consultants may perceive AI-driven automation as directly affecting specialized compliance and advisory functions that have traditionally required substantial professional expertise.

Cognitive adaptability also differed significantly across occupational groups, *F*_(4, 507)_ = 6.92, *p* < 0.001, η^2^ = 0.052. Accounting academics demonstrated the highest adaptability (*M* = 4.21, SD = 0.50), followed by finance managers (*M* = 4.06, SD = 0.54) and financial analysts (*M* = 4.04, SD = 0.55). These findings are consistent with the nature of academic and analytical professions, which typically involve continuous learning, knowledge acquisition, and engagement with emerging developments.

Collectively, the subgroup analyses reveal that demographic and professional characteristics exert meaningful influence on accounting professionals' psychological responses toward AI-enabled transformation. Although the structural model demonstrates how trust, anxiety, and adaptability influence perceptions of AI substitution, the inter-group analyses identify which groups are most likely to experience these psychological reactions. The results indicate that younger professionals, less experienced employees, and tax consultants constitute the groups most vulnerable to AI-related anxiety and uncertainty. Conversely, experienced professionals, finance managers, and accounting academics appear more confident and adaptable in responding to technological change.

These findings have important managerial implications. Organizations should avoid adopting uniform AI implementation strategies and instead develop targeted interventions that address the distinct concerns of different employee groups. Tailored training programs, mentoring initiatives, career-development support, and transparent communication regarding the role of AI may be particularly beneficial for younger and less experienced professionals. By recognizing the demographic and occupational heterogeneity of the accounting workforce, organizations can facilitate more effective AI adoption while minimizing resistance and technology-related anxiety.

### 4.3 Qualitative results and analysis

The qualitative phase was undertaken to complement, explain, and extend the quantitative findings by examining how accounting professionals interpreted the psychological and professional implications of AI-enabled transformation within future accounting frameworks in China. Semi-structured interviews were conducted with 25 participants, including auditors, accountants, finance managers, tax consultants, accounting academics, and specialists involved in AI-enabled financial systems. The interview data were analyzed using reflexive thematic analysis. As the study was conducted by a single author, all coding, categorization, and theme development were undertaken solely by the author. The previous reference to “two members of the research team” reflected wording from an earlier draft and has been removed to ensure consistency and transparency. To enhance trustworthiness, coding proceeded through iterative cycles of open coding, focused coding, and theme refinement, supported by a coding journal and audit trail documenting analytical decisions throughout the process.

Five major themes emerged from the analysis: (1) Trust Formation through Reliability, Explainability, and Verification; (2) Professional Identity Anxiety and Occupational Uncertainty; (3) Cognitive Adaptability as Professional Reinvention; (4) Human Judgment, Accountability, and the Boundaries of AI Substitution; and (5) The Emergence of a Human–AI Accounting Profession. While these themes broadly supported the quantitative findings, they also generated independent insights beyond the proposed hypotheses. In particular, participants consistently distinguished between task substitution and professional substitution, emphasized accountability as a uniquely human responsibility, and described AI adoption as a process of professional identity negotiation rather than merely technological acceptance. The qualitative findings therefore served two complementary functions. First, they contextualized the statistically significant relationships identified through the PLS-SEM model. Second, they revealed how accounting professionals construct trust, experience anxiety, redefine expertise, and negotiate responsibility in increasingly AI-enabled workplaces. Whereas the quantitative model explained the relationships among Psychological Trust, AI Anxiety, Cognitive Adaptability, and Perceived AI Substitution, the interviews illuminated the underlying meanings attached to those relationships and the conditions under which AI substitution was considered acceptable or unacceptable.

***Theme 1: trust formation through reliability, explainability, and verification***.

Consistent with the quantitative support for H1 and H4, trust emerged as a central factor shaping participants' perceptions of AI-enabled accounting systems. However, the interviews revealed that trust was not simply a positive attitude toward technology. Rather, trust was described as an earned professional judgment developed through repeated interaction with AI systems and continuous verification of their outputs.

Participants generally acknowledged the operational benefits of AI in processing large volumes of financial information, detecting anomalies, reconciling transactions, and generating analytical reports. Nevertheless, trust was rarely based on efficiency alone. Instead, respondents repeatedly emphasized explainability, transparency, and verifiability as prerequisites for relying on AI-generated outputs. A finance manager explained:

“*I trust AI when I can understand how it reached a conclusion. If I cannot explain the result to senior management, then I cannot confidently use it.”*

Similarly, a senior auditor observed:

“*The issue is not whether AI is fast. The issue is whether I can defend its recommendation during an audit review.”*

These statements suggest that trust formation in accounting is closely linked to professional accountability. Participants frequently noted that regulatory authorities, clients, and stakeholders hold human professionals—not algorithms—responsible for accounting outcomes. Consequently, trust was often described as confidence in the ability to verify AI recommendations rather than confidence in automation itself.

The interviews further revealed that trust varied according to task characteristics. Participants expressed high levels of trust when AI performed structured and rule-based functions such as invoice matching, transaction classification, reconciliation, and preliminary audit testing. However, trust declined substantially when tasks involved ambiguity, stakeholder interests, or contextual interpretation. As one tax consultant stated:

“*For routine tax calculations, AI is usually reliable. But when regulations are unclear or clients have unusual situations, professional judgment becomes more important than automation.”*

This distinction provides important insight into the positive relationship between Psychological Trust and Perceived AI Substitution (H1). Participants were willing to accept AI substitution when tasks were standardized, transparent, and easily verifiable. However, trust did not automatically translate into acceptance of occupation-wide replacement. Instead, trust operated within clearly defined task boundaries.

The interviews also clarified the positive influence of Psychological Trust on Cognitive Adaptability (H4). Participants who trusted AI systems were more willing to experiment with new technologies, participate in AI-related training, and redesign established workflows. Trust reduced uncertainty surrounding technological change and encouraged a learning-oriented mindset. As one accounting academic noted:

“*When professionals trust the technology, they stop asking whether they should use it and start learning how to use it effectively.”*

Therefore, trust functioned not only as a determinant of AI acceptance but also as a catalyst for professional adaptation. The findings indicate that trust in AI-enabled accounting environments is fundamentally rooted in explainability, accountability, and verification rather than blind confidence in technological capability.

***Theme 2: professional identity anxiety and occupational uncertainty***.

While the quantitative findings demonstrated a significant negative relationship between AI Anxiety and Perceived AI Substitution (H2) and between AI Anxiety and Cognitive Adaptability (H5), the interviews revealed that participants' concerns extended far beyond fears of job loss. The dominant concern was not immediate unemployment but the potential erosion of professional identity, expertise, and occupational value in an increasingly automated environment.

Participants frequently distinguished between losing a position and losing professional relevance. Several respondents explained that traditional accounting careers have historically been built upon the gradual acquisition of expertise through repetitive operational tasks such as bookkeeping, reconciliations, compliance reviews, and financial reporting. As these tasks become increasingly automated, participants questioned how future professionals would acquire the experiential knowledge that historically formed the foundation of professional judgment.

A junior accountant explained:

“*I am not worried that AI will replace me tomorrow. I worry that the skills I am learning today may not be the skills that make me valuable in five years.”*

Similarly, a tax consultant observed:explained:

“*The profession is changing so quickly that people are unsure what expertise will still matter. That uncertainty creates anxiety.”*

These statements suggest that AI anxiety is closely connected to professional identity formation. Participants frequently described accounting not merely as a collection of technical tasks but as a professional identity associated with expertise, credibility, and responsibility. Consequently, concerns regarding AI were often expressed in terms of occupational relevance rather than technological fear.

Notably, anxiety levels differed substantially across professional groups. Junior accountants and early-career professionals reported the highest levels of uncertainty because routine activities traditionally served as stepping stones toward advanced professional responsibilities. Participants feared that automation might eliminate opportunities to develop judgment, professional skepticism, and client-management skills.

A junior auditor remarked:explained:

“*If AI performs most of the basic audit procedures, where will young professionals learn the experience needed to become senior auditors?”*

By contrast, senior managers reported significantly lower anxiety. Rather than perceiving AI as a threat, they viewed it as a mechanism for enhancing organizational effectiveness and reallocating effort toward strategic activities. An experienced finance manager explained:

“*The value of accountants is shifting upward. The routine work is disappearing, but the need for interpretation and decision-making is increasing.”*

These findings extend the quantitative support for H2 by demonstrating that AI anxiety reflects concerns regarding employability, professional identity, expertise development, and future career trajectories rather than simple fears of technological replacement. Furthermore, the interviews provide a deeper explanation for H5. Participants experiencing high anxiety frequently described avoiding experimentation, delaying training participation, and resisting workflow changes. Anxiety therefore constrained adaptability by encouraging protective behaviors designed to preserve familiar professional roles.

***Theme 3: cognitive adaptability as professional reinvention***.

The quantitative findings demonstrated a significant positive relationship between Cognitive Adaptability and Perceived AI Substitution (H3) and confirmed the mediating role of Cognitive Adaptability between Psychological Trust and Perceived AI Substitution (H6). The interviews revealed that adaptability was not merely a willingness to learn new technologies. Rather, participants described adaptability as a process of professional reinvention involving the redefinition of what it means to be an accountant in an AI-enabled environment.

Participants consistently emphasized that future accounting professionals would require fundamentally different competencies than previous generations. Traditional expertise centered on transaction processing, compliance activities, and procedural accuracy was increasingly perceived as insufficient. Instead, respondents highlighted strategic thinking, analytical interpretation, communication skills, and technological literacy as emerging sources of professional value. An accounting academic stated:

“*The future accountant is not someone who records transactions. The future accountant is someone who interprets information generated by intelligent systems.”*

Similarly, a financial analyst remarked:

“*Adaptability is no longer about learning software. It is about redefining your role.”*

Participants exhibiting high levels of adaptability viewed technological transformation as an opportunity to expand professional capabilities. Rather than competing with AI systems, these individuals focused on identifying complementary skills that technology could not easily replicate. Many described transitioning from operational activities toward advisory, analytical, and decision-support responsibilities. A finance manager explained:

“*AI has reduced the time I spend checking numbers, which allows me to spend more time helping management make decisions.”*

Conversely, respondents with lower adaptability frequently described technological change as disruptive and overwhelming. Their concerns often stemmed from uncertainty regarding how existing expertise could be transferred into emerging work environments. Importantly, the interviews clarified the mediating mechanism identified in H6. Participants who trusted AI systems were more willing to engage in experimentation, training, and workflow redesign. These adaptive behaviors subsequently increased acceptance of AI-enabled substitution. Thus, trust encouraged adaptation, adaptation facilitated professional reinvention, and professional reinvention increased acceptance of AI integration.

The findings therefore extend H3 and H6 by demonstrating that cognitive adaptability represents a broader process of occupational transformation rather than merely technological learning.

***Theme 4: human judgment, accountability, and the boundaries of AI substitution***.

One of the strongest independent themes emerging from the interviews concerned accountability and professional responsibility. Participants consistently argued that the primary limitation of AI substitution is not computational capability but the inability of AI systems to assume accountability for decisions and their consequences.

Across all professional groups, respondents distinguished between producing recommendations and accepting responsibility for those recommendations. Although AI systems were widely regarded as capable of generating sophisticated analyses and identifying patterns within large datasets, participants emphasized that accountability remains fundamentally human. A senior auditor explained:

“*If an AI system makes a mistake, regulators do not question the algorithm. They question the auditor who approved the decision.”*

Similarly, a finance executive stated:

“*AI can generate recommendations, but it cannot accept legal responsibility for financial reporting.”*

Participants repeatedly highlighted situations involving ambiguity, competing stakeholder interests, ethical conflicts, and regulatory interpretation. These contexts were viewed as areas where professional judgment remained indispensable. One respondent observed:

“*Accounting decisions are rarely black and white. Sometimes the technically correct answer is not the most appropriate answer.”*

The interviews further revealed clear task-level boundaries of AI substitution. Participants generally accepted AI substitution in highly structured and rule-based activities such as transaction processing, reconciliations, invoice matching, compliance checking, and preliminary audit testing. However, they rejected the notion that AI could fully replace professional judgment in areas involving negotiation, ethical reasoning, risk assessment, or stakeholder communication.

A tax consultant remarked:

“*AI can prepare a tax analysis, but deciding how to advise a client still requires professional judgment and responsibility.”*

This distinction extends the quantitative findings by demonstrating that Perceived AI Substitution is fundamentally task-specific rather than profession-wide. Participants consistently separated substitution of activities from substitution of professional accountability. Consequently, acceptance of AI was highest when systems augmented human decision-making and lowest when automation appeared to challenge accountability and professional discretion.

***Theme 5: the emergence of a human–AI accounting profession***.

The final theme moved beyond simple notions of collaboration and focused on how participants envisioned the future evolution of accounting as a hybrid profession. Rather than describing AI and humans as separate actors working together, respondents frequently described the emergence of a new professional identity in which technological and human capabilities become deeply integrated.

Participants overwhelmingly rejected the idea that accounting would remain unchanged. Instead, they anticipated a profession characterized by continuous interaction between human expertise and intelligent systems. In this future environment, technical accounting knowledge would remain important, but success would increasingly depend on the ability to interpret AI-generated insights, supervise automated processes, and exercise judgment in complex situations. An accounting academic explained:

“*The profession is not disappearing. It is being redesigned.”*

Similarly, a financial analyst stated:

“*AI is becoming part of what accountants do every day. The future professional will be someone who understands both accounting and intelligent systems.”*

Respondents consistently argued that AI would assume responsibility for processing information, identifying anomalies, and generating predictive insights, while human professionals would focus on interpretation, ethical evaluation, strategic thinking, and stakeholder engagement. A finance manager summarized this transformation:

“*AI will replace tasks, but accountants who understand AI will replace accountants who do not.”*

This theme provides an important extension of the quantitative model. While H1–H6 explain the psychological mechanisms influencing AI acceptance, the interviews suggest that the ultimate outcome is not technological substitution alone but the emergence of a hybrid human–AI profession. Trust facilitates engagement with AI, anxiety influences willingness to adapt, and cognitive adaptability enables successful role transformation. Together, these psychological processes shape the evolution of accounting toward a model in which human expertise and intelligent systems function as integrated components of professional practice.

The qualitative findings reveal that future accounting frameworks are likely to be characterized not by complete occupational replacement but by substantial professional transformation. Participants consistently viewed AI as capable of substituting routine and analytical tasks, yet incapable of replacing accountability, ethical responsibility, contextual interpretation, and professional judgment. Consequently, the future of accounting is expected to involve the reconfiguration of professional roles rather than the elimination of the profession itself. Overall, the qualitative findings strongly support and expand the quantitative results obtained through PLS-SEM analysis. Psychological Trust enhanced acceptance of AI-enabled systems and encouraged adaptive learning behaviors, thereby supporting H1 and H4. AI Anxiety emerged as a significant source of resistance and reduced adaptability, consistent with H2 and H5. Cognitive Adaptability facilitated positive evaluations of AI substitution and mediated the influence of trust on acceptance, confirming H3 and H6.

More importantly, the interviews revealed that accounting professionals distinguish clearly between task substitution and occupational replacement. While participants recognized AI's substantial capability in automating repetitive and analytical activities, they consistently emphasized the continuing importance of ethical judgment, contextual interpretation, professional skepticism, and interpersonal communication. These findings directly reveal that that AI is unlikely to completely substitute human accountants in the foreseeable future. Rather, future accounting frameworks are expected to evolve through collaborative integration between intelligent technologies and human expertise, with psychological factors playing a decisive role in determining the success of this transformation.

### 4.4 Mixed-methods integration results

The integration of the quantitative and qualitative phases generated a more comprehensive understanding of accounting professionals' perceptions regarding AI substitution. The quantitative phase established the causal relationships among the study variables and identified generalizable patterns within the larger sample. Specifically, psychological trust emerged as a positive predictor of cognitive adaptability and perceived AI substitution, whereas AI anxiety functioned as a significant barrier. Cognitive adaptability further mediated the relationship between trust and AI substitution perceptions.

The qualitative phase complemented these findings by explaining the deeper psychological mechanisms underlying the statistical relationships. The interviews revealed how trust develops through perceptions of reliability and transparency, how anxiety is shaped by concerns regarding professional identity and job security, and how adaptability reflects continuous learning and role reconfiguration. Moreover, the qualitative evidence identified important situational boundaries and group differences that could not be captured through quantitative analysis alone. Grassroots employees exhibited greater anxiety toward AI-enabled transformation, senior managers emphasized strategic opportunities, and academic researchers adopted more balanced and reflective positions.

The integration of both phases leads to three overarching conclusions. First, AI is perceived as capable of substituting repetitive, standardized, and analytical accounting tasks but not of completely replacing human accountants. Second, uniquely human capabilities, including ethical judgment, contextual interpretation, interpersonal communication, and professional discretion, remain indispensable within accounting practice. Third, the effectiveness of AI implementation depends not solely on technological capability but also on the psychological readiness of accounting professionals. Trust facilitates acceptance and adaptability, whereas anxiety inhibits adjustment to technological change.

Collectively, these integrated findings suggest that the future of accounting will be characterized by collaborative human–AI relationships rather than technological displacement. The study therefore contributes to the literature by demonstrating that psychological factors determine how technological potential is translated into organizational reality and by highlighting the importance of trust-building, adaptive learning, and workforce development initiatives in facilitating sustainable AI transformation within the accounting profession.

## 5 Discussion

The present study examined how accounting professionals perceive the potential of AI to substitute accounting tasks and how psychological trust, AI anxiety, and cognitive adaptability shape these perceptions in China. The findings could not be interpreted as evidence that AI objectively replaces accountants. Rather, they reflect subjective evaluations formed by professionals operating within rapidly digitalizing accounting environments. The findings provide important theoretical and practical insights into how psychological mechanisms influence acceptance of AI-enabled accounting systems. More importantly, the results suggest that perceptions regarding AI substitution emerge through the interaction of psychological resources, emotional responses, adaptive capabilities, and professional contexts. Psychological trust and cognitive adaptability significantly enhance perceptions regarding AI substitution in accounting tasks, whereas AI anxiety negatively affects AI acceptance. The study further demonstrates that cognitive adaptability mediates the relationship between psychological trust and AI substitution perception.

One of the most significant findings of the study is the positive influence of psychological trust on perceptions regarding AI substitution within accounting systems. The results indicate that accounting professionals who perceive AI systems as reliable, transparent, and competent are more likely to support the substitution of human accounting tasks by intelligent technologies. However, the positive influence of psychological trust could not be interpreted as evidence that accounting professionals unconditionally endorse AI substitution. Rather, the findings suggest that trust reduces perceived uncertainty surrounding intelligent systems and increases willingness to delegate selected accounting functions. Trust therefore appears to facilitate acceptance of task redistribution rather than support for complete occupational replacement. This finding strongly supports Trust Theory, which argues that trust reduces uncertainty and increases individuals' willingness to depend on automated systems under conditions of technological complexity (Mayer et al., 1995). The result is also consistent with the Technology Acceptance Model (TAM), which suggests that technologies perceived as useful and dependable positively influence adoption behavior (Davis, 1989).

The finding aligns with prior studies emphasizing the critical role of trust in AI adoption. (Glikson and Woolley 2020) reported that trust significantly influences users' willingness to rely on AI systems across organizational settings. Similarly, (Choung et al. 2023) found that trust positively affects acceptance of AI-based decision-support technologies because users perceive trusted systems as accurate and beneficial. Within accounting contexts, (Issa et al. 2016) argued that professionals are more likely to utilize intelligent auditing systems and predictive analytics when they trust AI-generated outputs. The present study extends these findings by demonstrating that trust not only influences AI usage intentions but also shapes broader perceptions regarding the future substitution of human accounting functions by AI systems. Importantly, the qualitative findings suggest that trust itself is multidimensional, encompassing confidence in algorithmic competence, transparency, explainability, and accountability. Professionals appeared willing to accept AI substitution primarily when intelligent systems generated outputs that could be interpreted, justified, and aligned with professional standards.

The positive role of trust may be explained by the increasing sophistication of AI-enabled accounting systems in China. Intelligent accounting technologies are now capable of automating bookkeeping, auditing, taxation, fraud detection, and predictive financial analysis with substantial accuracy and efficiency. Accounting professionals who trust these systems are more likely to perceive AI as capable of performing repetitive and analytical accounting tasks more effectively than humans. However, the findings also imply that trust remains essential because accounting activities involve highly sensitive financial decisions requiring reliability and accountability.

The study further found that AI anxiety negatively influences perceptions regarding AI substitution within accounting frameworks. Accounting professionals experiencing fear, uncertainty, and discomfort regarding AI technologies demonstrated lower acceptance of AI-driven accounting systems. This finding supports previous research emphasizing the negative psychological consequences of technological anxiety. (Wang and Wang 2019) argued that AI anxiety generates resistance toward intelligent systems because individuals fear technological uncertainty and loss of professional control. Similarly, (Li and Huang 2020) and (Yu et al. 2025) found that AI-related fears significantly reduce employees' willingness to engage with intelligent technologies within organizations.

The findings also support earlier accounting studies indicating that automation anxiety remains a major barrier to digital transformation within financial professions. (Kokina and Davenport 2017) noted that accounting professionals frequently perceive AI systems as threats to career stability because intelligent technologies increasingly perform tasks traditionally executed by humans. Likewise, (Sutton et al. 2016) suggested that the growing automation of accounting processes creates concerns regarding skill obsolescence and reduced professional relevance.

The negative relationship between AI anxiety and AI substitution perception can be explained through concerns regarding job displacement and professional identity. In China's rapidly digitalizing accounting sector, professionals may fear that intelligent systems will substitute junior and routine accounting roles, thereby reducing employment opportunities. Such concerns create psychological resistance toward AI-enabled accounting transformation. The finding therefore highlights that technological advancement alone is insufficient for successful AI integration unless organizations simultaneously address employees' emotional and psychological concerns. Similarly, AI anxiety reflects more than discomfort with technology. The qualitative evidence suggests that anxiety is rooted in concerns regarding professional identity, employability, and future relevance within increasingly digital workplaces. Consequently, resistance to AI may represent an effort to preserve occupational meaning rather than simple opposition to technological innovation.

Another important finding of the study is the positive effect of cognitive adaptability on AI substitution perception. Cognitive adaptability emerged as a strong psychological mechanism translating favorable attitudes into acceptance of AI-enabled transformation. This finding suggests that adaptive capacity may buffer professionals against automation-related uncertainty by enabling them to redefine their roles and acquire competencies suited to human–AI collaboration. The results indicate that accounting professionals capable of adjusting cognitive strategies and learning new technological systems are more willing to accept AI integration within accounting functions. This finding supports (Haynie et al. 2010), who argued that cognitive adaptability enables individuals to respond effectively to dynamic and uncertain environments. Adaptable individuals are generally more capable of embracing innovation, modifying work practices, and integrating advanced technologies into professional activities.

The finding is also consistent with prior research emphasizing the importance of adaptability in technological transformation. (Armstrong and Mahmud 2008) found that individuals possessing strong adaptive learning capabilities are more likely to accept innovative systems and technological change. Similarly, (Park and Woo 2022) reported that cognitive adaptability positively influences AI adoption within financial service sectors because adaptable professionals perceive intelligent technologies as opportunities rather than threats.

Within accounting environments, cognitive adaptability becomes particularly important because AI implementation requires continuous learning, technological flexibility, and skill transformation. Professionals with higher adaptability are more capable of understanding intelligent systems, modifying accounting workflows, and collaborating with AI technologies. The finding therefore suggests that adaptability represents a critical capability for future accountants operating within AI-driven financial ecosystems. The study also revealed that psychological trust positively influences cognitive adaptability. Accounting professionals who trust AI systems were more likely to demonstrate adaptive learning behaviors and technological flexibility. This finding supports previous studies indicating that trust encourages openness toward technological experimentation and innovation adoption (Gefen et al., 2003). When professionals perceive AI systems as reliable and beneficial, they become more willing to invest cognitive effort in learning and integrating intelligent technologies into accounting practices.

Conversely, AI anxiety negatively affected cognitive adaptability. Professionals experiencing fear and uncertainty regarding AI technologies demonstrated lower willingness to adapt cognitively to technological transformation. This finding aligns with (Thatcher and Perrewé 2002), who found that technological anxiety reduces learning confidence and innovation acceptance. Anxiety may discourage accounting professionals from engaging with AI-related training and technological experimentation, thereby limiting their adaptability toward intelligent accounting systems.

One of the most significant contributions of the study is the identification of cognitive adaptability as a mediator between psychological trust and AI substitution perception. The mediation analysis demonstrated that trust enhances AI substitution perception indirectly by improving professionals' adaptability toward technological change. This finding extends previous technology adoption research by explaining the psychological mechanism through which trust influences AI acceptance. Importantly, the persistence of the direct relationship between psychological trust and AI substitution perception alongside the significant indirect effect suggests a partial rather than complete mediation process. Trust therefore appears to influence acceptance through two complementary pathways: directly by reducing uncertainty and strengthening confidence in AI systems, and indirectly by encouraging adaptive learning, experimentation, and competence development. Consequently, cognitive adaptability transforms positive psychological trust into active technological engagement while trust itself retains an independent influence on perceptions of AI substitution.

The findings also provide important implications regarding whether AI can completely substitute human accountants in the future. Although the results indicate substantial support for AI substitution in repetitive and analytical accounting tasks, the findings simultaneously suggest that human cognitive capabilities remain indispensable in several accounting functions. AI systems may excel in processing large datasets, detecting anomalies, generating predictive analytics, and automating routine operations. However, accounting also involves ethical reasoning, professional skepticism, contextual judgment, strategic thinking, and interpersonal communication that AI systems may not fully replicate. This finding supports prior studies arguing that AI is more likely to complement rather than completely substitute human accountants. (Moll and Yigitbasioglu 2019) emphasized that future accounting professionals will increasingly collaborate with intelligent technologies rather than compete against them. Similarly, (Davenport and Kirby 2016) argued that human expertise remains essential in technologically advanced environments because intelligent systems cannot fully substitute human creativity, ethical judgment, and emotional intelligence.

An important implication emerging from both the quantitative and qualitative findings is that AI substitution in accounting could be understood at the task level rather than the occupational level. Participants acknowledged AI's effectiveness in automating repetitive, rule-based, and analytical activities, while simultaneously emphasizing the continued importance of professional judgment, ethical reasoning, stakeholder communication, and strategic interpretation. These findings align more closely with a human–AI complementarity perspective than with a full substitution perspective. Accordingly, the future accounting profession may involve the redistribution of tasks between humans and intelligent systems rather than the elimination of human expertise.

The supplementary inter-group analyses further indicate that accounting professionals do not constitute a homogeneous population in their responses to AI-enabled transformation. Female respondents reported higher levels of AI anxiety despite exhibiting comparable levels of trust and adaptability, suggesting that concerns regarding technological disruption may vary according to perceptions of career uncertainty. Similarly, younger professionals and those with limited experience exhibited lower trust and higher anxiety, whereas respondents with greater professional experience demonstrated stronger trust and adaptability. Differences across occupational roles also emerged, with finance managers viewing AI more strategically and tax consultants expressing comparatively greater concern regarding automation. These findings suggest that psychological responses to AI are shaped by career stage, professional experience, and occupational responsibilities, indicating that organizational interventions should avoid one-size-fits-all approaches.

The findings suggest that perceived AI substitution is not solely a function of beliefs regarding technological capability. Rather, it reflects broader psychological processes through which professionals interpret technological change. Trust in AI represents confidence in algorithmic competence and explainability, AI anxiety reflects concerns regarding job insecurity and threats to professional identity, and cognitive adaptability captures occupational adjustment within evolving work environments. Consequently, perceptions regarding AI substitution should be understood as responses to anticipated changes in professional roles rather than evaluations of technological efficiency alone. This interpretation extends traditional technology acceptance perspectives by highlighting the psychology of human–AI collaboration in professional contexts. Accounting professionals appear to negotiate the boundaries between human expertise and machine intelligence while redefining their occupational identities in response to technological disruption. Thus, AI acceptance within accounting is fundamentally embedded in processes of identity preservation, adaptation, and collaboration.

The findings could be interpreted in light of the exploratory nature of the PAIS construct. The study does not claim to measure actual AI substitution, technological capability, accounting accuracy, or future occupational replacement. Rather, the construct captures accounting professionals' perceptions and expectations regarding the potential role of AI within future accounting frameworks. Accordingly, significant relationships between psychological trust, AI anxiety, cognitive adaptability, and PAIS should be understood as relationships influencing subjective perceptions of AI substitution rather than objective assessments of AI performance. The results therefore contribute primarily to understanding how professionals psychologically interpret technological transformation and evolving human–AI work arrangements. This distinction is important because perceptions of substitution may differ substantially from actual technological capabilities. Individuals may overestimate or underestimate AI's future role depending on psychological, organizational, and contextual factors. Consequently, the present findings contribute to literature on occupational perceptions of AI rather than providing evidence concerning the technical feasibility of replacing accountants.

The Chinese context of the study further contributes to understanding AI-accounting transformation within emerging digital economies. China's rapid investment in AI development and intelligent financial systems has accelerated accounting automation across industries. However, the findings suggest that psychological readiness remains equally important as technological readiness in ensuring successful AI integration. Accounting professionals' trust, adaptability, and emotional responses significantly shape how AI transformation unfolds within organizational settings. Therefore, sustainable AI implementation requires organizations not only to invest in intelligent technologies but also to cultivate trust, support adaptive learning, and address the diverse concerns experienced by different groups of accounting professionals.

## 6 Conclusion

### 6.1 Summary of findings

The present study examined accounting professionals' perceptions of AI substitution in future accounting frameworks by investigating the roles of psychological trust, AI anxiety, and cognitive adaptability among accounting professionals in China using a mixed-method approach integrating PLS-SEM and qualitative analysis. The findings showed that psychological trust positively influenced perceptions of AI substitution, whereas AI anxiety negatively affected acceptance of AI-enabled accounting systems. Cognitive adaptability emerged as a significant positive determinant and partially mediated the relationship between trust and AI substitution perception. This suggests that trust enhances acceptance both directly by reducing uncertainty and indirectly by strengthening professionals' adaptive capacity toward technological change.

The integrated findings further revealed that perceptions of AI substitution are multidimensional. Trust reflected confidence in AI competence, transparency, and explainability; AI anxiety encompassed concerns regarding professional identity, employability, and future relevance; and cognitive adaptability represented continuous learning and role adjustment within AI-enabled environments. Accordingly, perceived AI substitution reflected broader psychological responses to changing professional roles rather than evaluations of technological capability alone. Although AI demonstrated substantial potential to automate repetitive, analytical, and data-intensive accounting activities, the findings suggest that it cannot fully substitute human accountants because ethical reasoning, professional judgment, strategic interpretation, emotional intelligence, and interpersonal communication remain uniquely human strengths. The evidence therefore supports task-level substitution and human–AI complementarity rather than complete occupational replacement.

The supplementary analyses further indicated that psychological trust, anxiety, and adaptability differed across age groups, professional experience, and occupational positions, suggesting that accounting professionals do not respond uniformly to AI transformation. Within the Chinese context, successful AI integration depends not only on technological advancement but also on professionals' psychological readiness and adaptive capabilities. Consequently, organizations pursuing AI-enabled accounting transformation must address both technological efficiency and human factors to achieve sustainable digital change.

### 6.2 Theoretical contributions

The study contributes significantly to existing literature on artificial intelligence and accounting in several ways. First, the study extends the Technology Acceptance Model (TAM) and Trust Theory by integrating psychological trust, AI anxiety, and cognitive adaptability into the AI-accounting adoption framework. While prior studies primarily focused on technological usefulness and organizational efficiency, the present research emphasizes the psychological mechanisms influencing AI substitution perceptions within accounting professions. In doing so, it reconceptualizes perceived AI substitution as a psychologically embedded process through which professionals negotiate evolving occupational boundaries rather than as a purely technological judgment.

Second, the study contributes to AI adoption literature by identifying cognitive adaptability as a mediating variable between psychological trust and AI substitution perception. This contribution advances theoretical understanding regarding how trust influences AI acceptance through adaptive cognitive processes. The findings demonstrate that adaptability functions as a critical psychological bridge connecting trust with technological engagement and acceptance behavior. At the same time, the observed partial mediation indicates that trust retains an independent influence on AI substitution perceptions beyond its indirect effect through adaptability. This finding highlights the coexistence of affective mechanisms based on confidence and developmental mechanisms based on adaptation.

Third, the study enriches accounting technology literature by incorporating emotional and cognitive dimensions into discussions regarding intelligent accounting transformation. Previous accounting studies largely emphasized automation efficiency and operational outcomes, whereas the current study highlights the importance of trust, anxiety, and adaptability in shaping professionals' responses toward AI-enabled accounting systems. The findings further support a human–AI complementarity perspective by demonstrating that professionals distinguish between the substitution of specific accounting tasks and the replacement of the accounting profession itself.

Fourth, the study contributes methodologically by adopting a mixed-method research design integrating quantitative PLS-SEM analysis with qualitative thematic insights. This methodological integration enhances analytical depth and provides a comprehensive understanding regarding psychological and technological aspects of AI substitution within accounting frameworks. The qualitative findings not only contextualized the statistical relationships but also revealed the mechanisms, situational boundaries, and professional differences underlying those relationships.

Finally, the study contributes contextually by focusing on China, one of the world's leading AI-driven economies. The findings provide valuable insights into how accounting professionals within rapidly digitalizing emerging economies perceive AI transformation and technological substitution, thereby extending the geographical scope of AI-accounting scholarship beyond predominantly Western contexts

### 6.3 Practical implications

The findings of the study provide several important practical implications for accounting firms, policymakers, educational institutions, and AI developers. For accounting organizations, the study highlights the importance of building psychological trust in AI-enabled accounting systems. Organizations should ensure transparency, reliability, explainability, and accuracy of AI technologies to enhance professionals' confidence in intelligent accounting systems. Providing clear information regarding how AI systems operate and generate decisions can significantly reduce uncertainty and increase acceptance among employees.

The study also emphasizes the need to address AI anxiety among accounting professionals. Organizations should implement reskilling and upskilling programs to help employees adapt to technological transformation and reduce fears regarding job displacement. Training initiatives focusing on AI literacy, digital accounting competencies, and technological collaboration can improve professionals' confidence and reduce psychological resistance toward intelligent systems. Given the observed differences across demographic and occupational groups, interventions should be tailored to the specific concerns and developmental needs of employees at different career stages and professional positions rather than relying on uniform implementation strategies.

The significant role of cognitive adaptability suggests that accounting firms should promote adaptive learning cultures within organizations. Continuous professional development programs, AI-related workshops, mentoring initiatives, and technological training opportunities can strengthen employees' adaptability toward evolving accounting technologies. Encouraging innovation-oriented organizational cultures may further facilitate successful AI integration within accounting operations.

For policymakers, the findings indicate the importance of developing balanced AI governance frameworks that support technological advancement while protecting workforce sustainability. Policymakers should encourage educational and professional institutions to redesign accounting curricula by incorporating AI, data analytics, digital finance, and intelligent auditing competencies to prepare future accountants for AI-driven financial environments.

Educational institutions should revise accounting education programs to include interdisciplinary learning combining accounting knowledge with artificial intelligence, machine learning, data analytics, and digital transformation skills. Developing adaptive and technologically competent accounting graduates who are capable of collaborating effectively with intelligent systems will become increasingly essential in future accounting ecosystems.

For AI developers and technology providers, the study suggests that intelligent accounting systems should be designed with user-centered approaches emphasizing explainability, ethical accountability, transparency, and collaborative functionality. AI technologies should complement human accountants rather than attempt complete replacement, thereby creating sustainable human–AI partnerships within accounting professions.

### 6.4 Limitations and future scope

The present study has several limitations that provide opportunities for future research. First, the study focused exclusively on accounting professionals in China, which may limit the generalizability of the findings to other countries with different technological, cultural, and regulatory environments. Future studies may conduct cross-country comparative analyses to examine how cultural and institutional differences influence perceptions regarding AI substitution in accounting frameworks and whether the psychological mechanisms identified in the present study operate similarly across contexts.

Second, the study adopted a cross-sectional research design, which captures respondents' perceptions at a single point in time. Since AI technologies and accounting systems continue to evolve rapidly, future longitudinal studies may provide deeper insights into how psychological trust, anxiety, adaptability, and perceptions of AI substitution change over time with increasing AI exposure and professional experience.

Third, the study examined only psychological trust, AI anxiety, and cognitive adaptability as determinants of AI substitution perception. Future research could incorporate additional variables such as ethical concerns, organizational support, technological readiness, perceived job insecurity, digital leadership, AI literacy, professional identity, and explainability perceptions to develop a more comprehensive understanding of AI adoption in accounting professions.

Fourth, although the present study utilized mixed methods, future studies may employ experimental designs, case studies, diary methods, or industry-specific investigations to explore how different AI applications influence accounting roles, adaptive processes, and professional identity in various organizational contexts. Future research may also examine how human–AI collaboration evolves across different accounting specializations and whether the balance between task substitution and professional complementarity changes as intelligent technologies continue to advance.

Fifthly, an important limitation concerns the use of the self-developed Perceived AI Substitution Scale (PAIS). Although the scale demonstrated satisfactory reliability, convergent validity, and discriminant validity, it remains an exploratory measure developed specifically for this study. The current research provides only preliminary evidence regarding its psychometric properties. Additional validation efforts involving independent samples, longitudinal designs, alternative occupational groups, exploratory and confirmatory factor analyses, and criterion-related validation are required before PAIS can be considered a fully established measurement instrument. Therefore, conclusions involving PAIS should be interpreted with appropriate caution. Finally, although several procedural and statistical measures were implemented to mitigate common method bias, including respondent anonymity, confidentiality assurances, Harman's single-factor test, and full collinearity VIF assessment, the study relied on self-reported data collected from the same respondents at a single point in time. Consequently, the possibility of residual common method variance cannot be entirely ruled out. Future research may strengthen validity by employing longitudinal, time-lagged, multi-source, or experimental research designs.

## Data Availability

The datasets presented in this article are not readily available because anonymized quantitative data and de-identified qualitative excerpts supporting the conclusions of this study are available from the corresponding author upon reasonable request for academic purposes, subject to the protection of participant confidentiality Requests to access the datasets should be directed to ymike4354@gmail.com.
