# Supplementary material for: Exploring perceived AI substitution in future accounting frameworks: the role of psychological trust, anxiety, and cognitive adaptability
Source: Front Psychol. 2026 Jul 20;17:1875355. doi: 10.3389/fpsyg.2026.1875355 (PMC13429411; doi:10.3389/fpsyg.2026.1875355)
Supplement: Supplementary file 1 [file Data_Sheet_1.docx]

**Appendix**

Semi-Structured Interview Protocol

Title: Semi-Structured Interview Protocol for Exploring Accounting Professionals' Perceptions of AI-Enabled Transformation

Introduction to Participants

Thank you for agreeing to participate in this interview. The purpose of this discussion is to better understand accounting professionals' experiences and perceptions regarding the increasing use of artificial intelligence in accounting practice. There are no right or wrong answers. We are interested in your personal experiences and opinions. Participation is voluntary, and you may decline to answer any question or withdraw from the interview at any time without any consequences. With your permission, the interview will be audio-recorded to ensure accurate transcription and analysis. All information will remain confidential and will be reported only in anonymized form.

Module 1: Experiences with AI Technologies in Accounting Practice

1. Can you describe your experience with AI-enabled technologies in your current professional role?
2. What types of AI applications or intelligent systems do you use most frequently?
3. How have these technologies changed your day-to-day accounting activities?
4. Can you provide examples of tasks that have been significantly affected by AI implementation?
   Probes:
   • When did your organization begin adopting these technologies?
   • How often do you interact with AI systems?

Module 2: Opportunities and Challenges of AI Integration

1. What benefits do you believe AI brings to accounting practice?
2. What challenges or limitations have you encountered?
3. How has AI influenced efficiency, accuracy, and decision-making?
4. What organizational barriers affect successful AI implementation?
   Probes:
   • Technical challenges.
   • Resource constraints.
   • Organizational support and training.

Module 3: Professional Identity, Job Security, and Career Trajectories

1. Do you believe AI threatens traditional accounting roles? Why or why not?
2. How has AI affected your perceptions of job security?
3. What accounting functions do you believe remain uniquely human?
4. How do you foresee accounting careers evolving in the future?
   Probes:
   • Professional judgment.
   • Ethical decision-making.
   • Human oversight responsibilities.

Module 4: Learning, Adaptation, and Cognitive Responses

1. How have you adapted to technological changes introduced by AI?
2. What new skills have become necessary?
3. Have you experienced anxiety, uncertainty, or resistance related to AI adoption?
4. What factors facilitate successful adaptation?
   Probes:
   • Training experiences.
   • Self-directed learning.
   • Organizational learning support.

Module 5: Future Relationship Between Human Accountants and Intelligent Systems

1. How do you envision the future relationship between accountants and AI technologies?
2. Do you anticipate collaboration or substitution?
3. What safeguards should organizations establish when implementing AI?
4. What recommendations would you offer to accounting professionals preparing for an AI-enabled future?
   Probes:
   • Ethical governance.
   • Transparency and explainability.
   • Human–AI complementarity.

Closing Questions

1. Is there anything else you would like to share regarding AI and the future of accounting?
2. Do you have any recommendations for organizations, educators, or policymakers?

Closing Statement

Thank you for your valuable contribution. Your insights will help develop a deeper understanding of how accounting professionals experience and respond to AI-enabled transformation. All information provided will remain confidential and will be used exclusively for academic research purposes.
